# CoCeOx-PVP Catalyst for Effective CO-SCR in the Presence of O_2_

**DOI:** 10.3390/molecules30051133

**Published:** 2025-03-01

**Authors:** Yuansong Zhou, Fengyu Gao, Lei Yi, Junyi Wang, Honghong Yi, Xiaolong Tang

**Affiliations:** Beijing Key Laboratory of Resource-Oriented Treatment of Industrial Pollutants, School of Energy and Environmental Engineering, University of Science and Technology Beijing, Beijing 100083, China; zhouys@ustb.edu.cn (Y.Z.); 15973617828@163.com (L.Y.); wjyhjgc@126.com (J.W.); yhhtxl@163.com (H.Y.)

**Keywords:** CO-SCR, polyvinylpyrrolidone, oxygen vacancy, CoCeOx-PVP, O_2_-containing environment

## Abstract

In an O_2_-containing environment, achieving efficient selective catalytic reduction of nitrogen oxides (NOx) by carbon monoxide (CO) using non-noble metal catalysts remains a formidable challenge. To balance the catalytic oxidation of CO and the catalytic reduction of NOx, we need to develop a catalyst with strong reductibility and weak oxidizability for the CO selective catalytic reduction of NOx (CO-SCR) reaction in the presence of O_2_. In this study, we synthesized the CoCeOx-PVP catalyst via a coprecipitation method and employed various characterization techniques, including BET, SEM, XRD, Raman, XPS, H_2_-TPR, and O_2_-TPD. The analysis results indicate that the addition of polyvinylpyrrolidone (PVP) alters the surface structure of the catalyst, increases the particle size, and enhances the concentration of surface oxygen vacancies. These structural effects facilitate electron circulation and accelerate the migration of oxygen species, thereby improving the catalytic reduction performance of the catalyst and increasing the conversion rate of NOx. At 250 °C and with 5 vol% O_2_, the conversion rates of NOx and CO can attain 98% and 96%, respectively, accompanied by a remarkable N_2_ selectivity of 99%. Following a sustained reaction period of 6 h, the conversion efficiencies of both NOx and CO remain above 95%. However, during extended testing periods, as the oxygen vacancies are progressively occupied by O_2_, the oxygen vacancies generated through the reduction of NO with CO fall short of sustaining the CO-SCR reaction over the long haul. Subsequently, the oxidation reactions of NO and CO come to dominate, resulting in a decline in the NOx conversion rate. Notably, the CO conversion rate still maintains 100% at this point. Based on the results of in situ diffuse reflectance infrared Fourier transform spectroscopy (in situ DRIFTS) experiments, we proposed a reaction mechanism for the CO-SCR process over the CoCeOx-PVP catalyst under O_2_-containing conditions. This study provides an effective strategy for the application of non-noble metal catalysts in the field of CO-SCR. Although maintaining long-term activity of the catalyst remains a challenge in the presence of O_2_, the catalyst in this study exhibits a slower deactivation rate compared to traditional non-noble metal catalysts.

## 1. Introduction

Nitrogen oxides (NOx), emitted primarily from stationary sources (such as power plants), mobile sources (such as vehicle exhaust emissions), and other industrial sources, can trigger a series of severe environmental problems, including acid rain, ozone depletion, enhanced greenhouse effects, and photochemical smog [1,2,3,4]. They also pose significant threats to human health, particularly increasing the risk of respiratory diseases. Given the increasing global regulation of NOx emissions, the issue of NOx reduction has become a focal point of intense research interest [5,6,7]. Ammonia selective catalytic reduction (NH_3_-SCR) technology is widely recognized as one of the effective means to eliminate NOx [8,9,10]. However, this technology faces several challenges in practical applications, such as high operational costs due to NH_3_ slip, potential toxic hazards, and catalyst poisoning caused by the formation of ammonium sulfate ((NH_4_)_2_SO_4_) or ammonium bisulfate (NH_4_HSO_4_) [11]. Notably, industrial flue gases from steel plants, coking plants, and mobile source exhaust often contain carbon monoxide (CO). As a reducing gas, it can replace ammonia to selectively catalyze the reduction of NOx to nitrogen (N_2_), a process known as CO-SCR [12,13]. CO-SCR technology not only removes NOx but also eliminates the harmful gas CO simultaneously, making it a promising method for controlling both pollutants [14,15,16].

Significant progress toward developing a more efficient CO-SCR catalyst has been achieved in recent years. Particularly, cobalt oxide (CoOx) and cerium oxide (CeOx) have shown excellent catalytic performance in the catalytic reduction of NO by CO (NO-CO reaction). Co_3_O_4_ is known as an active and prevalent catalyst for CO catalytic oxidation at low temperature [17,18]. It has been reported that Co_3_O_4_ and Co_3_O_4_-MOx binary oxides exhibit broad application potential in CO oxidation and NO-CO reactions due to their multivalent characteristics (such as the interconversion between Co^3+^ and Co^2+^, which generates active oxygen species) and strong CO adsorption capabilities [19,20,21]. CeO_2_, as a type of rare earth oxide, has been widely used as an important component or structural and electronic promoter in heterogeneous catalysts due to its excellent oxygen storage capacity (OSC) [22,23,24]. Through the rapid and reversible redox reaction between Ce^4+^ and Ce^3+^, molecular oxygen can be easily directed to the surface of CeO_2_, enabling it to exhibit superior N_2_ selectivity in the presence of O_2_. However, CeO_2_ is limited by its poor thermal stability and small specific surface area, leading to insufficient activity in the catalytic reduction of NO by CO (NO-CO) reaction [25,26]. Furthermore, studies have found that there exists a catalytic synergistic effect between Co and Ce oxides in various reactions, and Co_3_O_4_-CeO_2_ binary oxide exhibits excellent catalytic performance as a CO-SCR catalyst in the presence of O_2_. For example, Deng et al. compared the CO-SCR catalytic activity of different metals doped with CeO_2_ and found that CeCoOx performed best [27]. Savereide et al. prepared CoOx/CeO_2_ catalysts with CeO_2_ nanorods as the carrier, which exhibited excellent performance in NO-CO reaction, attributed to the rough surface and abundant oxygen vacancy defects of the CeO_2_ nanorod carrier [28]. Liu et al. employed the ball-milling method to induce the formation of interfacial oxygen vacancies (IOVs) at the composite heterogeneous interface of the Co_3_O_4_-CeO_2_ catalyst. This catalyst exhibited a NOx conversion rate of 94% and N_2_ selectivity of 100% within the temperature range of 200–350 °C [29]. However, when there is excessive O_2_ in the reaction atmosphere (O_2_/CO ratio is more than 1), the CoCeOx catalyst will preferentially adsorb the excessive O_2_, lowering the NO conversion N_2_ selectivity and, in severe cases, potentially causing catalyst deactivation [30].

In recent years, researchers have discovered that polyvinyl pyrrolidone (PVP), serving as a precipitation buffer, can effectively slow down the reaction rate during the coprecipitation process, enabling the active component structure to form slowly and orderly. This characteristic allows PVP to precisely control the growth process of catalyst crystals, thereby regulating the particle size and ultimately optimizing the redox performance of the catalyst [31,32]. Additionally, PVP possesses the ability to act as an electron donor, influencing the electronic structure of the catalyst and enhancing its activity by promoting the generation of superoxide species [33,34].

In this work, we successfully synthesized CoCeOx-PVP catalyst, CoCeOx catalyst, CoOx catalyst, and CeOx catalyst using the coprecipitation method. By introducing PVP, we effectively modified the surface structure of the catalysts, controlled their size, and subsequently influenced the surface electronic structure, promoting the formation of more surface oxygen vacancies. When applied as catalysts for the selective catalytic reduction of CO (CO-SCR), we comprehensively evaluated the NOx conversion, CO conversion, N_2_ selectivity, and stability of CoCeOx-PVP catalysts within a temperature range of 150 to 400 °C in the presence of 5 vol% O_2_. To delve deeper into the physicochemical properties of these catalysts, we employed various characterization techniques, including scanning electron microscopy (SEM), X-ray diffraction (XRD), N_2_ physisorption with Brunauer–Emmett–Teller (BET) surface area measurement, Raman spectroscopy, X-ray photoelectron spectroscopy (XPS), hydrogen temperature-programmed reduction (H_2_-TPR), and oxygen temperature-programmed desorption (O_2_-TPD). The in situ diffuse reflectance infrared Fourier transform spectroscopy (in situ DRIFTS) analysis reveals the reaction pathway of CO-SCR and analyzed the specific impact of O_2_ on the CO reduction of NO reaction. This study not only unveils the unique catalytic mechanism of CoCeOx-PVP catalysts in the CO-SCR reaction under O_2_-containing conditions but also provides new insights and strategies for the application of non-noble metal catalysts in the field of CO-SCR.

## 2. Results and Discussion

### 2.1. Physical Properties (N_2_ Physisorption, SEM, XRD, and Raman)

#### 2.1.1. N_2_ Physisorption

**Figure 1:**
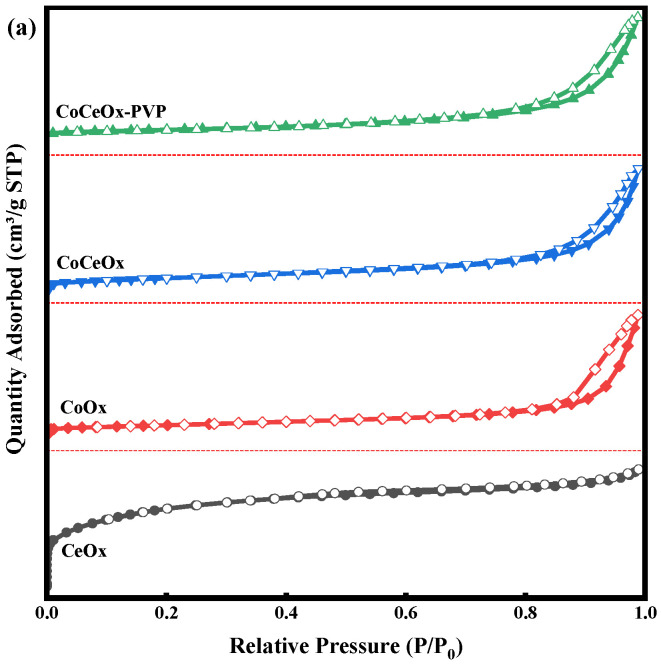

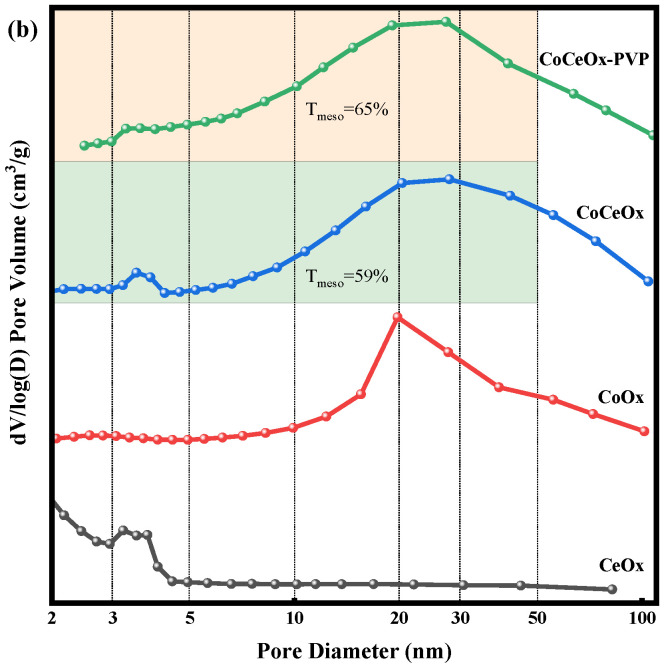
Characterization of N_2_ physisorption. (**a**) N_2_ adsorption–desorption isotherms and (**b**) pore size distribution.

The pore size distribution and surface area of the catalysts were tested by N_2_ physical adsorption. Figure 1a shows the N_2_ adsorption–desorption profiles. All catalysts, with the exception of the CeOx catalyst, display isotherms characterized by an H3 hysteresis loop in the relative pressure range of 0.7–1.0, indicating the presence of a wide range of pores, especially the mesoporous structures, and each catalyst possesses a pore loop network by the IUPAC definition. The pore size distributions are calculated by the BJH method with the results shown in Figure 1b. It can be observed that the pore diameters of CoCeOx-PVP, CoCeOx, and CoOx catalysts are around 10–50 nm, while the pore diameters of the CeOx catalyst are concentrated from 2–5 nm, indicating a dominating existence of mesopores.

The surface areas are calculated by the BET method and presented in Table 1, together with total pore volume and average pore diameter. Among them, the CoCeOx catalyst exhibits the highest total pore volume, with its specific surface area and pore diameter falling between those of the CoOx and CeOx catalysts. It is speculated that the mutual doping of CoOx and CeOx may result in the formation of a solid solution-like structure, altering the original pore structure of the catalyst. This structural change leads to the generation of more mesopores, thereby increasing the pore volume and exposing more active sites. On the other hand, the CoCeCox-PVP catalyst displays the largest average pore diameter but the smallest specific surface area and a pore volume lower than that of the CoCeOx catalyst. This indicates that the addition of PVP tends to promote particle agglomeration, resulting in an enlargement of particle size, which leads to a decrease in specific surface area. Nevertheless, this agglomeration effect modifies the pore architecture of the catalyst, leading to an expansion of pore size and an elevation in the proportion of mesopores (Tmeso = 65%). Consequently, this may facilitate the ingress of a greater number of reactant molecules into the pores, thereby enhancing the contact opportunities between reactants and the catalyst. The obtained data correspond closely with the discussions of Figure 1.

#### 2.1.2. SEM Observation

**Figure 2:**
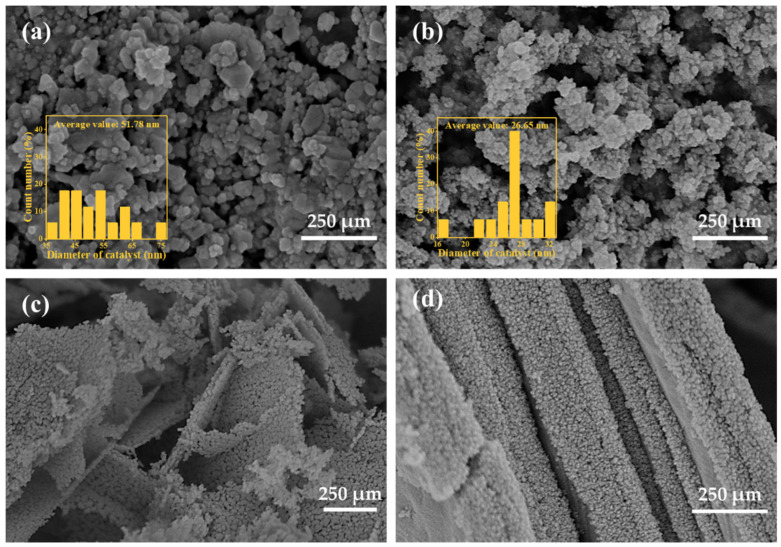
The SEM images of (**a**) CoCeOx-PVP, (**b**) CoCeOx, (**c**) CoOx, and (**d**) CeOx.

The SEM images of CoCeOx-PVP, CoCeOx, CoOx, and CeOx catalysts are shown in Figure 2. As can be seen, all the catalysts have high dispersibility. The morphology of the CoOx catalyst shown in Figure 2c is composed of densely arranged crystal particles of varying sizes, forming a single-layer structural morphology. As shown in Figure 2d, the CeOx catalyst has a flaky morphology, which is formed by the homogeneous and tightly combined multilayer grains. Through the presentations in Figure 2a,b, we can clearly observe that both the CoCeOx-PVP catalyst and CoCeOx catalyst are composed of aggregates of particles with similar sizes. The surface of the aggregates exhibits a distinct rough texture. The formation of this rough surface is due to the mutual doping and tight bonding of two highly dispersed oxides, resulting in uneven textures on the material’s surface. As revealed in a previous study, CeO_2_ modification is beneficial for the enhancement of dispersity, thermal stability, and smaller crystallite size of catalysts, which can promote the adsorption and conversion of the reactants [35,36,37]. By the size distributions of the CoCeOx-PVP catalyst and CoCeOx catalyst, it is found that the latter show a small size when PVP is not added, with an average diameter value of about 26.65 nm. However, after the addition of PVP, the particle boundaries become more distinct and the size increases significantly, and the average diameter value of the CoCeOx-PVP catalyst can reach 51.78 nm. The results indicate that PVP slows down the reaction rate of the coprecipitation process, resulting in a slower nucleation rate of crystals and an increase in the grain size of the catalyst [38].

#### 2.1.3. XRD and Raman Analysis

**Figure 3:**
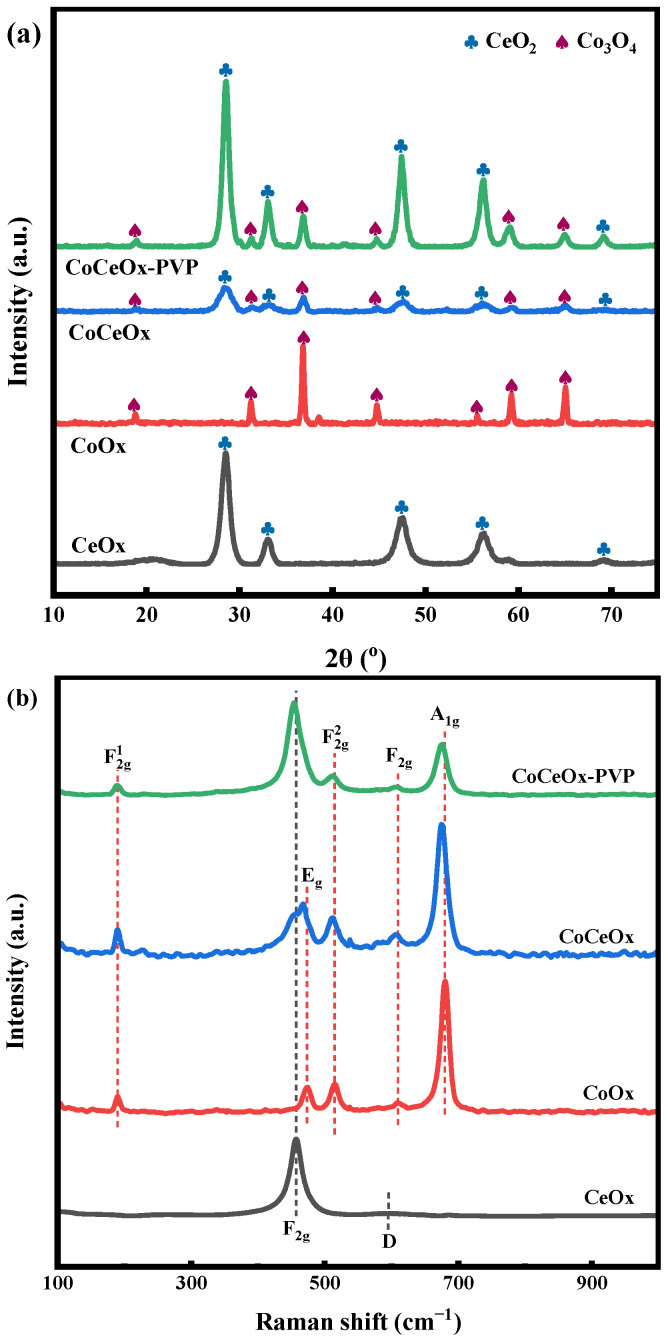
The XRD pattern (**a**) and Raman spectra (**b**) of all the catalysts.

The XRD diagram is then tested to identify the crystal phase on the catalysts as shown in Figure 3a. Over the CoCeOx-PVP catalyst, the planes of cubic-phased Co_3_O_4_ (PDF#42-1467) could be observed at 2*θ* values of 18.8°, 31.3°, 36.9°, 44.8°, and 65.0°, respectively. The planes of cubic CeO_2_ (fluorite structure, PDF#81-0792) could also be observed at 2*θ* values of 28.5°, 33.1°, 47.5°, 56.2°, 59.0°, and 69.1°, respectively. The XRD peak position patterns of the CoCeOx catalyst and the CoCeOx-PVP catalyst are consistent, but the peak height of Co_3_O_4_ is significantly lower than that of CeO_2_, indicating the successful in situ construction of Co_3_O_4_ and CeO_2_. Furthermore, the surfaces of both the CoCeOx-PVP catalyst and CoCeOx catalyst are dominated by CeO_2_, with the content of Co_3_O_4_ being lower than that of CeO_2_ [39]. However, XRD diffraction peaks of the CoCeOx-PVP catalyst are sharper, with narrower full width at half maximum (FWHM), indicating that the addition of PVP results in increased grain size, better crystal crystallinity, and reduced internal strain, which is consistent with the data calculation results (Table 2). Moreover, as shown in Figure 3a, the addition of PVP significantly enhances the diffraction peak intensity of the CoCeOx-PVP catalyst. This is attributed to PVP’s precise control over the nucleation rate, which facilitates the uniform and orderly growth of CoCeOx [40]. This conclusion is in agreement with the SEM images.

To further investigate the effect of species migration on the metal–oxygen arrangement and lattice defects in the catalyst, Raman spectroscopy has been employed [41]. The Raman spectra of different catalysts are shown in Figure 3b. For the catalyst CoOx, five peaks can be detected, corresponding to the $F_{2g}^{1}$ (~190 cm^−1^), $E_{g}$ (~474 cm^−1^), $F_{2g}^{2}$ (~515 cm^−1^), $F_{2g}$ (~609 cm^−1^), and $A_{1g}$ (~687 cm^−1^) phonon modes of Co_3_O_4_ spinel oxides. The $F_{2g}^{1}$, $F_{2g}^{2}$, and $F_{2g}$ peaks can be assigned to the tetrahedral sites (Co^2+^O_4_), whereas the $E_{g}$ and $A_{1g}$ peaks are assigned to the octahedral sites (Co^3+^O_6_) within the Co_3_O_4_ lattice, respectively. For the catalyst CeOx, the Raman spectra present a characteristic peak at around ~457 cm^−1^, corresponding to the $F_{2g}$ vibration mode of octahedral local symmetry around the cubic fluorite structure of CeO_2_. The broad shoulder at ~696 cm^−1^ could be attributed to the oxygen vacancy (OV) and defect-induced (D) vibrations, which are related to Ce^3+^-O^−^-Ce^4+^-type defect sites [29,39]. For the CoCeOx catalyst, five Raman peaks corresponding to Co_3_O_4_ can be observed, while the characteristic peak ($F_{2g}$) of CeO_2_ is obscured and nearly undetectable. This may be due to the interaction between Co_3_O_4_ and CeO_2_, resulting in the suppression of the structural characteristics of CeO_2_. After the addition of PVP, strong characteristic peaks ($F_{2g}$) of CeO_2_ can be observed in the CoCeOx-PVP catalyst, while the intensity of the Co_3_O_4_ Raman peaks decreases. This may be caused by a stronger interaction at the interface between Co_3_O_4_ and CeO_2_ due to lattice contraction/extension and surface reconstruction, which provides the motivation for the migration of O [29,42]. This is consistent with the results from XRD. Moreover, the $E_{g}$ peak disappeared in the CoCeOx-PVP catalyst, which may be associated with Co^3+^ ↔ Co^2+^ transitions, indicating that active Co sites are more easily reduced to Co^2+^ species [42].

### 2.2. Chemical Properties (XPS, H_2_-TPR, and O_2_-TPD)

#### 2.2.1. XPS

**Figure 4:**
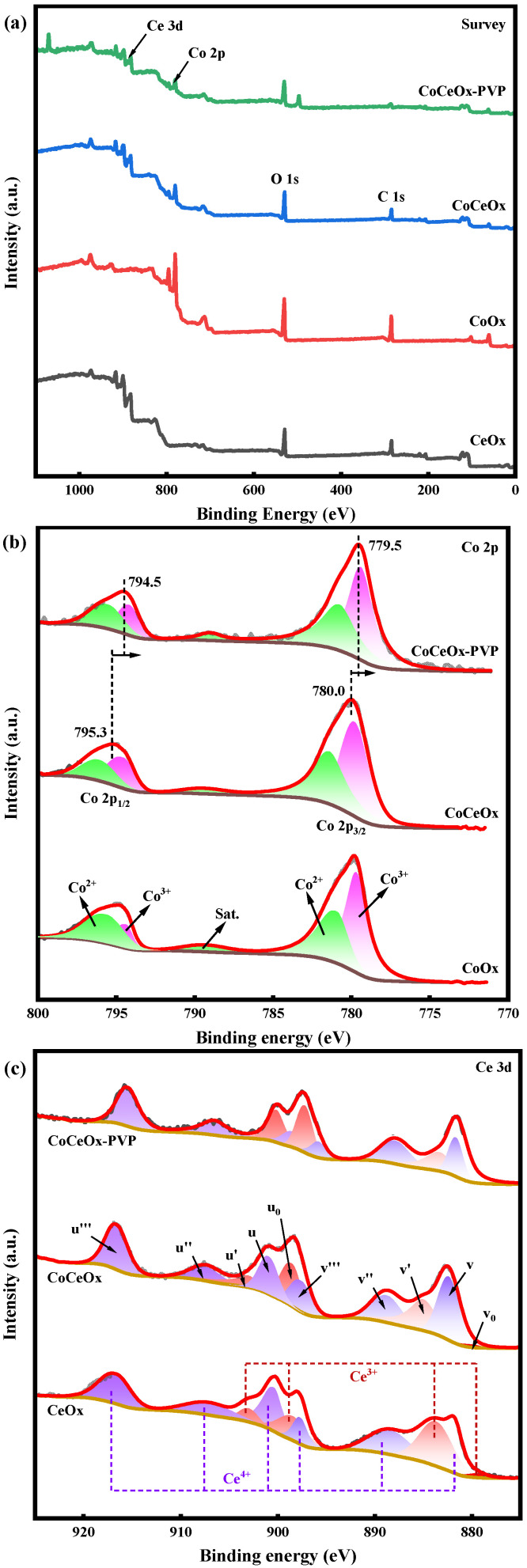

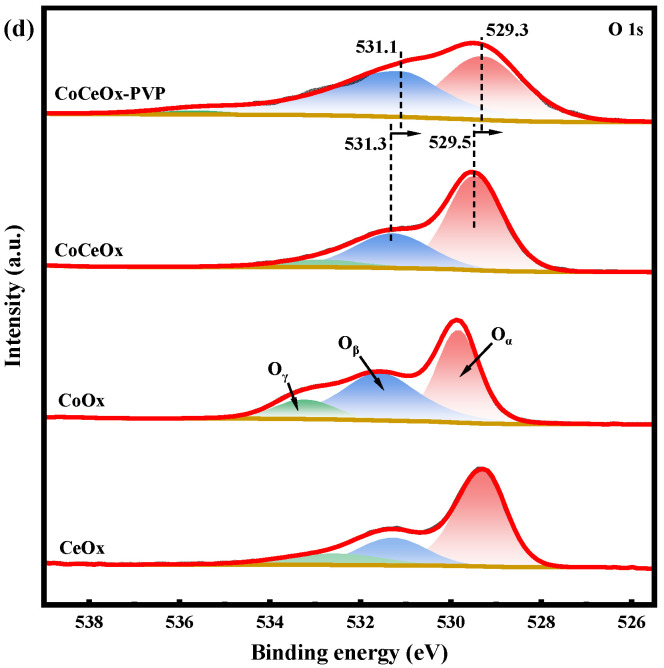
XPS spectra. (**a**) Survey, (**b**) Co 2p, (**c**) Ce 3d, (**d**) O 1s.

XPS spectra of the catalysts CoCeOx-PVP, CoCeOx, CoOx, and CeOx are taken to investigate the chemical valence state of the main components with the results shown in Figure 4. The survey spectra are presented in Figure 4a, indicating the presence of Co, Ce, and O, as well as C, which is used as the reference for binding energy calibration. As shown in Figure 4b, the XPS spectra of Co 2p exhibit two distinct spin–orbit peaks of Co 2p_3/2_ and Co 2p_1/2_. For the CoCeOx-PVP catalyst, CoCeOx catalyst, and CoOx catalyst, five peaks are seen with binding energy at 779.5 eV (2p_2/3_) and 794.5 eV (2p_1/2_), assigned to Co^3+^ species, and 780.9 eV (2p_2/3_) and 795.6 eV (2p_1/2_) plus shake-up satellite peaks, assigned to Co^2+^ species. As summarized in Table 3, the Co^2+^/Co ratio decreases following the order of CoCeOx-PVP > CoCeOx > CoOx. It reveals that, when Co is doped with Ce, electron transfer occurs between Co^3+^ and Ce^3+^, resulting in a shift to the right of the redox equilibrium (Co^3+^ + Ce^3+^ ↔ Co^2+^ + Ce^4+^). When PVP is added to the catalyst, this electron transfer process is further facilitated [29]. Moreover, the ratio of Co^2+^/Co of CoCeOx-PVP is determined to be 49.58, which is higher than that of the CoCeOx (45.63) and indicative of more surface oxygen vacancies [43,44,45]. It is particularly noteworthy that, compared to the CoCeOx catalyst, the Co 2p_3/2_ and Co 2p_1/2_ peaks on the CoCeOx-PVP catalyst exhibit a shift towards lower binding energy. This discovery implies that the addition of PVP can facilitate the formation of Co species with lower chemical valence states, which may have a positive impact on enhancing the catalytic redox reaction.

The XPS spectra of Ce 3d for the CoCeOx-PVP catalyst, CoCeOx catalyst, and CeOx catalyst are shown in Figure 4c. They can be deconvoluted into ten peaks, and the two spin–orbital peaks related to 3d_3/2_ and 3d_5/2_ are marked as u and v, respectively. Among them, the peaks labeled as v_0_, v′, u_0_, and u′ at 880.1, 883.6, 898.5, and 903.0 eV correspond to Ce^3+^ species, while the binding energy peaks v′, v″, v′″, u, u″, and u′″ are located at 881.7, 888.8, 897.6, 900.4, 907.0, and 916.6 eV and belong to Ce^4+^ species. The calculated contents of Ce^3+^ and Ce^4+^ by estimating the peak area are summarized in Table 3. It indicates that three catalysts are almost identical, with Ce mainly existing in the form of Ce^4+^, while the presence of Ce^3+^ is evidence for the generation of oxygen vacancies. Among these catalysts, the CoCeOx-PVP catalyst exhibits a higher concentration of surface Ce^3+^. The increase in Ce^3+^ can also be ascribed to redox equilibrium (Co^3+^ + Ce^3+^ ↔ Co^2+^ + Ce^4+^) shifting to the left due to the enhanced electron transfer between Co^2+^ and Ce^4+^ [44,46]. However, both Co^2+^ and Ce^3+^ species are increased simultaneously on the CoCeOx-PVP catalyst, which may be attributed to the oxygen deficiency at the interface of Co and Ce.

Concerning oxygen in the catalysts, XPS spectra in Figure 4d could be deconvoluted into three peaks and ascribed to lattice oxygen O*_α_* at 529.3 eV (lattice oxygen combines with the metal cations), surface chemisorbed oxygen species and vacancy oxygen O*_β_* at 531.3 eV (chemical reaction forms the oxygen vacancy, oxygen in the lattice as M-O-M, which may later be vacated as M-□-M during catalytic reactions), and surface hydroxyl oxygen O*_γ_* at 533.2 eV (surface oxygen, such as oxygen contamination, -OH, -CO_3_, and adsorbed O_2_) [47,48], respectively. The fraction of each oxygen species could be calculated according to the area ratios of these peaks and they are summarized in Table 3.

Based on the comparison among the CoCeOx catalyst, CoOx catalyst, and CeOx catalyst, the decreasing order of O*_α_* concentration is as follows: CeOx > CoCeOx > CoOx. Conversely, the decreasing order of O*_β_* concentration is the opposite: CoOx > CoCeOx > CeOx. The lower surface O*_α_*/O ratio and higher surface O*_β_*/O ratio on the CoCeOx-PVP catalyst than on the CoCeOx catalyst imply a lack of some surface lattice oxygen atoms but more abundant surface chemisorbed oxygen species and oxygen vacancies in the former. In general, it has been frequently considered that O*_β_* is essential for the catalytic activity since oxygen vacancy plays a key role in the oxygen transfer during the redox reaction. Therefore, the CoCeOx catalyst with the addition of PVP possesses more oxygen vacancies and exhibits better catalytic performance, which is consistent with the analysis results of the XPS spectra for Co 2p and Ce 3d. The positions of oxygen species follow an order of CoCeOx-PVP < CoCeOx (binding energy). Lower binding energies of oxygen on CoCeOx-PVP may also facilitate the redox cycle of oxygen during catalytic reactions.

#### 2.2.2. H_2_-TPR and O_2_-TPD

**Figure 5:**
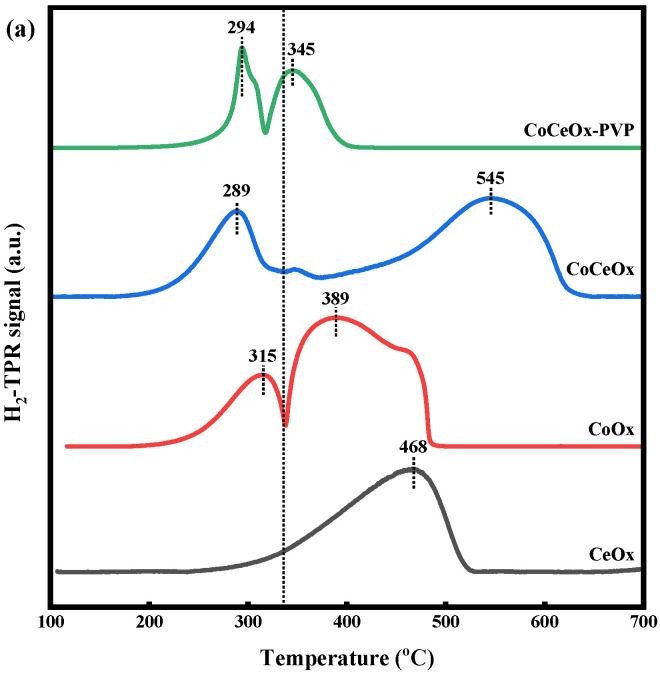

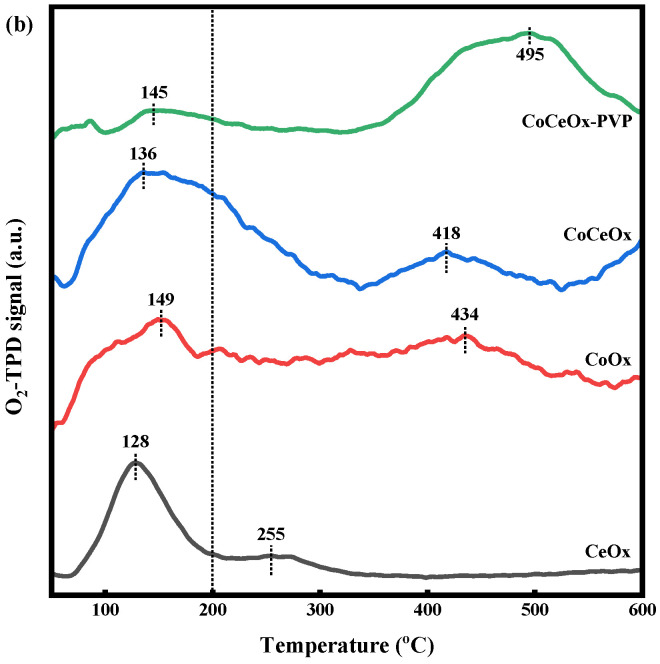
(**a**) H_2_-TPR profiles and (**b**) O_2_-TPD profiles.

To delve deeper into the reducibility of catalysts, we conduct hydrogen temperature-programmed reduction (H_2_-TPR) analysis on the CoCeOx-PVP catalyst, CoCeOx catalyst, CoOx catalyst, and CeOx catalyst within a broad temperature range from 100 °C to 700 °C as shown in Figure 5a. In the H_2_-TPR spectrum, the first peak observed at a temperature range of 289–315 °C is attributed to the reduction of Co^3+^ to Co^2+^. For the CoCeOx-PVP catalyst, the subsequent peak at 345 °C corresponds to the reduction of Co^2+^ to Co within the structure of Co^2+^-OV-Co^2+^ or Co^2+^-OV-Ce^4+^ (where OV denotes oxygen vacancy) [49,50,51]. In the case of the CoCeOx catalyst, the second peak at 545 °C signifies the coexistence of the reduction of Co^2+^ to Co and the surface capping oxygen (O^2−^) of CeO_2_ [40,52]. For the CoOx catalyst, the second peak at 389 °C also signifies the reduction of Co^2+^ to Co, with the area ratio of these two peaks being approximately 1:3, which aligns well with the theoretical hydrogen consumption expected during the reduction of Co_3_O_4_. For the CeOx catalyst, the peak at 468 °C belongs to the surface capping oxygen of CeO_2_. Comparing the CoCeOx catalyst to the CoOx and CeOx catalysts, it is observed that the second reduction peak of the CoCeOx catalyst shifts to a higher temperature after the doping process with Co and Ce, suggesting the generation of strong interaction between Co_3_O_4_ and CeO_2_ in this catalyst. In contrast, after the addition of PVP, the second reduction peak of the CoCeOx-PVP catalyst moves to a lower-temperature region than that of the CoCeOx catalyst, with no observable reduction peak of surface capping oxygen of CeO_2_. This indicates an enhanced reducibility of the CoCeOx-PVP catalyst, and the absence of surface capping oxygen indicates the probable formation of oxygen vacancies [53]. Meanwhile, as shown in Table 4, the total H_2_ consumption of the CoCeOx-PVP catalyst is less than that of the CoCeOx catalyst. This aligns well with the disappearance of the reduction peak for surface capping oxygen of CeO_2_ on the CoCeOx-PVP catalyst. Furthermore, this also demonstrates that the phenomenon of oxygen migration occurs between Co_3_O_4_ and CeO_2_ upon the addition of PVP, potentially contributing to the formation of oxygen vacancies.

To further understand the oxygen vacancies (OVs) on the catalyst surface, an O_2_-TPD analysis is performed. In general, the oxygen species present on the catalyst surface undergo the following processes: O_2_(ads)→$O_{2}^{-}$(ads)→O^−^(ads)→O^2−^, which are classified as physical adsorbed oxygen (50–200 °C), surface chemisorbed adsorbed oxygen (200–600 °C), and lattice oxygen in bulk (600–800 °C) [54,55], respectively. Physical adsorption of O_2_ from the surface is mostly removed by inert gases, surface chemisorbed oxygen is associated with OVs, and the adsorbed oxygen species are mainly involved in redox processes. Considering the temperature range of the CO-SCR reaction, which typically falls between 150 °C and 450 °C, lattice oxygen in bulk is largely uninvolved in the reaction [26]. Therefore, the focus is on comparing the oxygen desorption behavior of different catalysts at temperatures ranging from 50 to 600 °C in Figure 5b. The desorption peaks of all the catalysts are divided into two subpeaks. The peak at 128–145 °C is attributed to the desorption of physically adsorbed oxygen, and the peak at 255–495 °C belongs to the surface chemisorbed adsorbed oxygen. Among them, the peak area of chemically adsorbed oxygen for the CoCeOx-PVP catalyst is significantly greater than that of physically adsorbed oxygen and also higher than that of other catalysts, suggesting the presence of abundant OVs. This may be related to the addition of PVP, which alters the particle size and surface pore structure of the catalyst, thereby exposing more active sites. In addition, as shown in Table 4, based on the content of surface chemisorbed oxygen species calculated by the peak area, the surface chemisorbed oxygen amount of CoCeOx-PVP is the highest, which further confirms the presence of a large number of OVs in this catalyst.

### 2.3. CO-SCR Performances of the Catalysts

**Figure 6:**
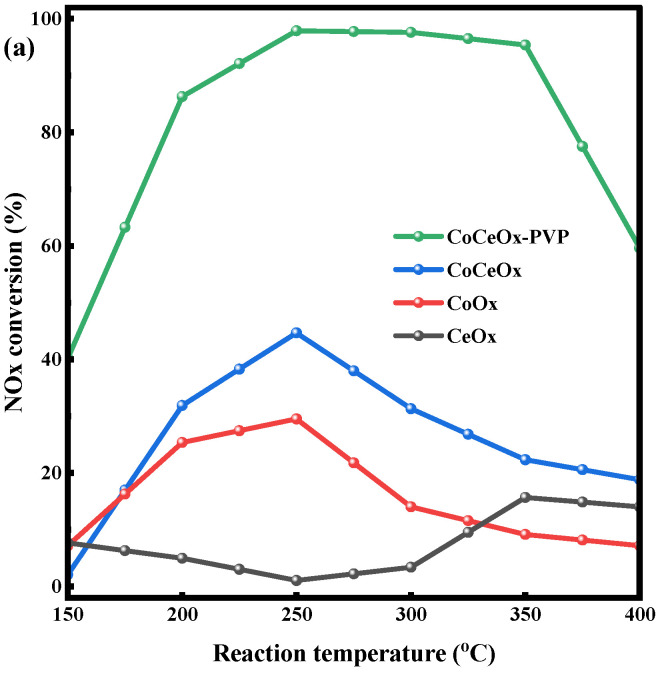

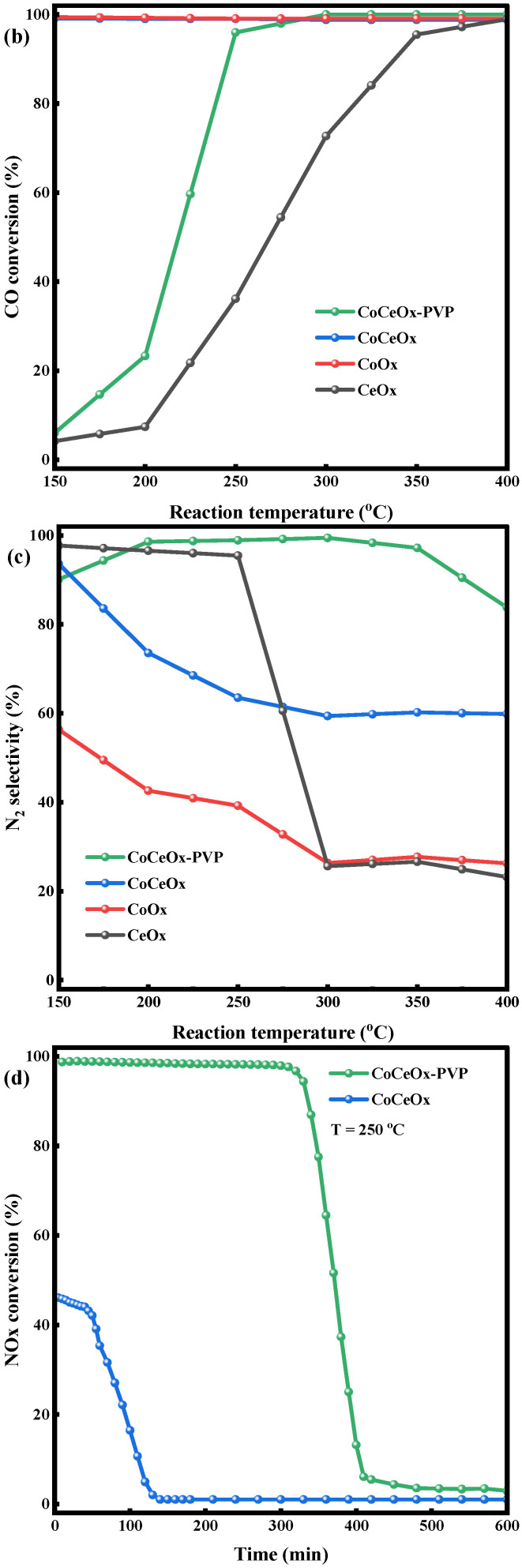

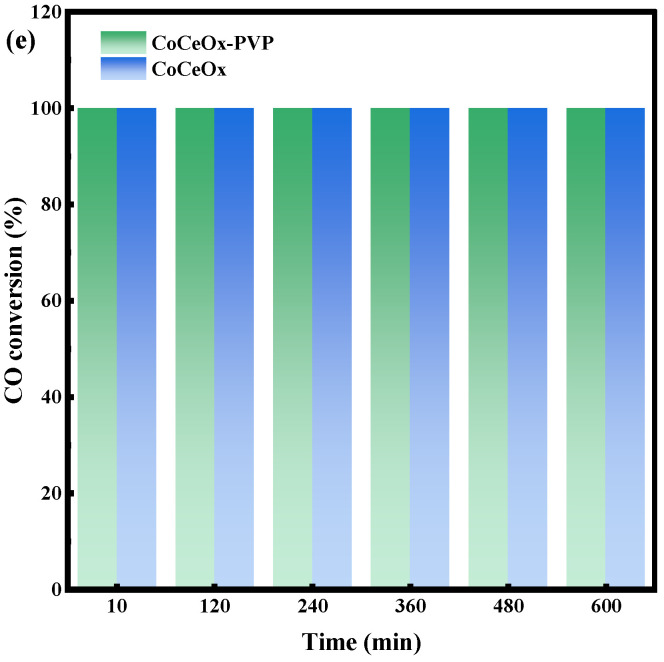
(**a**) NOx conversion, (**b**) CO conversion, and (**c**) N_2_ selectivity of different catalysts as a function of reaction temperature in CO-SCR. (**d**) Long-term stability of NOx conversion, (**e**) Long-term stability of CO conversion over CoCeOx-PVP and CoCeOx catalysts (Reaction conditions: [NO] = 1000 ppm, [CO] = 3000 ppm, [O_2_] = 5 vol%, and N_2_ as balanced gas, GHSV = 20,000 h^−1^).

Figure 6a provides a detailed illustration of the catalytic performance of the CoCeOx-PVP catalyst, CoCeOx catalyst, CoOx catalyst, and CeOx catalyst in the CO selective catalytic reduction (CO-SCR) reaction under an atmosphere containing 5 vol% oxygen. As the temperature increases, except for the CeOx catalyst, the NOx conversion of the catalysts exhibits a volcano-type tendency. At each temperature point (150–400 °C), the NOx conversion of CoCeOx-PVP catalysts is higher than that of the other three catalysts, particularly reaching nearly 100% NOx conversion within the temperature range of 250–350 °C. The CoCeOx and CoOx catalysts exhibit similar trends in NOx conversion, but with lower conversion rates compared to the CoCeOx-PVP catalyst. The CeOx catalyst shows the lowest catalytic activity, with an NOx conversion of only around 15% above 350 °C. The CO conversion reaches 100% above 150 °C for CoCeOx and CoOx catalysts, while for the CoCeOx-PVP catalyst, a reaction temperature above 250 °C is required for the CO conversion to reach 100%. The CeOx catalyst requires a reaction temperature above 350 °C for this to occur (Figure 6b). Notably, for the CoCeOx-PVP catalyst, 100% N_2_ selectivity is achieved in the temperature range of 200–350 °C (Figure 6c). Therefore, the CoCeOx-PVP catalyst with PVP addition exhibits the best NO catalytic performance. Based on the physicochemical characterization of the catalysts, this may be due to the fact that the CoCeOx-PVP catalyst is prone to forming a large number of oxygen vacancies (OVs), possessing strong reducibility, and thus facilitating the reduction reaction between NO and CO. In contrast, the CoOx and CoCeOx catalysts have lower NOx conversion but higher CO conversion, which is because, apart from the catalytic reduction reaction between NO and CO, CO oxidation also occurs, and Co_3_O_4_ can catalyze the oxidation of CO by O_2_. Notably, compared to most reported catalysts, CoCeOx-PVP exhibits excellent catalytic performance in CO-SCR (Table 5).

We further examined the long-term stability of the CoCeOx-PVP catalyst and the CoCeOx catalyst at 250 °C. As shown in Figure 6d, the CoCeOx-PVP catalyst initially showed approximately 100% NOx conversion and maintained it above 95% for 6 h, but it then dropped to 5% in the following hour. The CoCeOx catalyst initially reached 46% NOx conversion, maintained it above 40% for 1 h, and then dropped it to 2% in the next hour. However, CO conversion of both catalysts remained at 100% (Figure 6e). This indicates that, during the NO and CO reaction process, the CO-SCR reaction occurs simultaneously with the CO oxidation reaction, but as the reaction continues, the CO oxidation reaction becomes dominant, blocking the CO-SCR reaction. The addition of PVP cannot change this trend but can slow down the occurrence of this phenomenon. There may be two reasons for this situation: Firstly, the concentration of O_2_ is much higher than that of CO, causing CO to be directly oxidized to CO_2_ and thus failing to participate in the CO-SCR reaction; secondly, the oxygen vacancies in the catalyst are gradually filled by O_2_, which hinders the cyclic path of CO reducing NO.

### 2.4. In Situ DRIFTS Measurements

**Figure 7:**
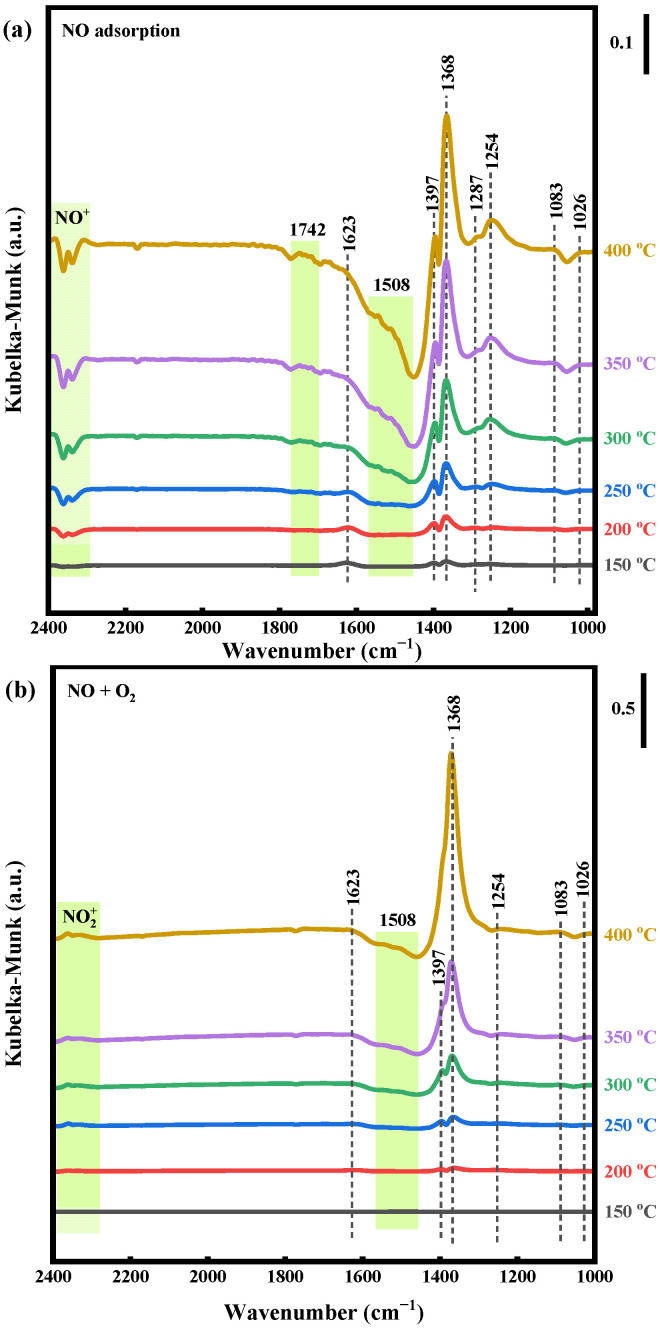

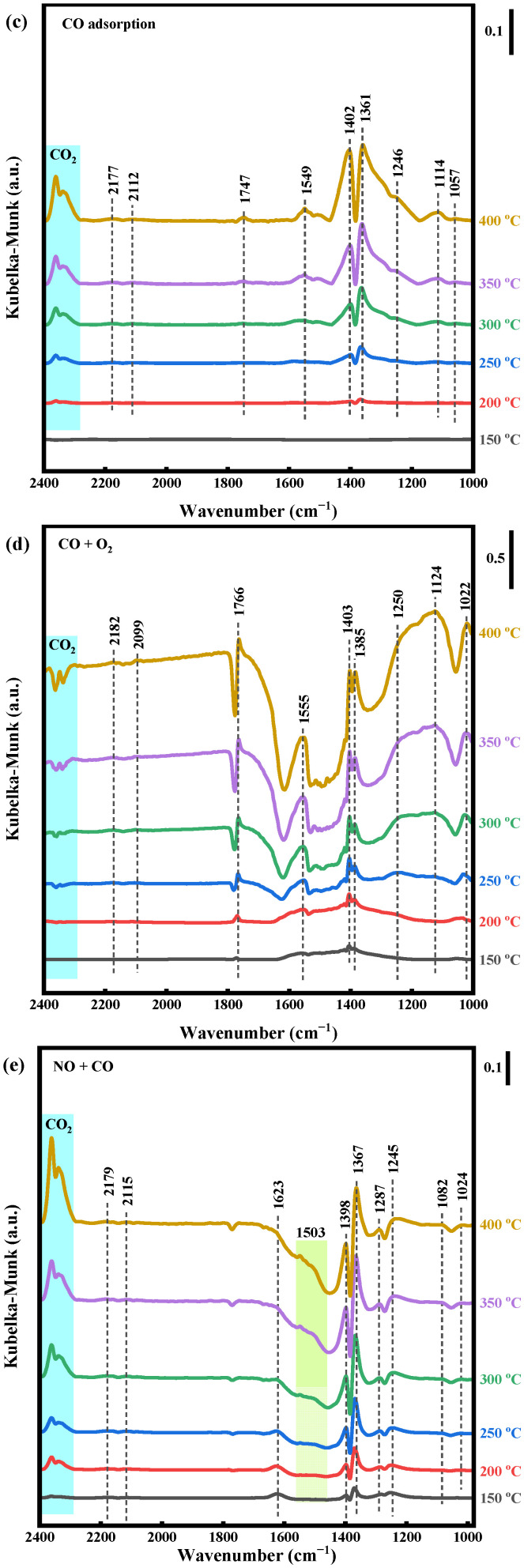

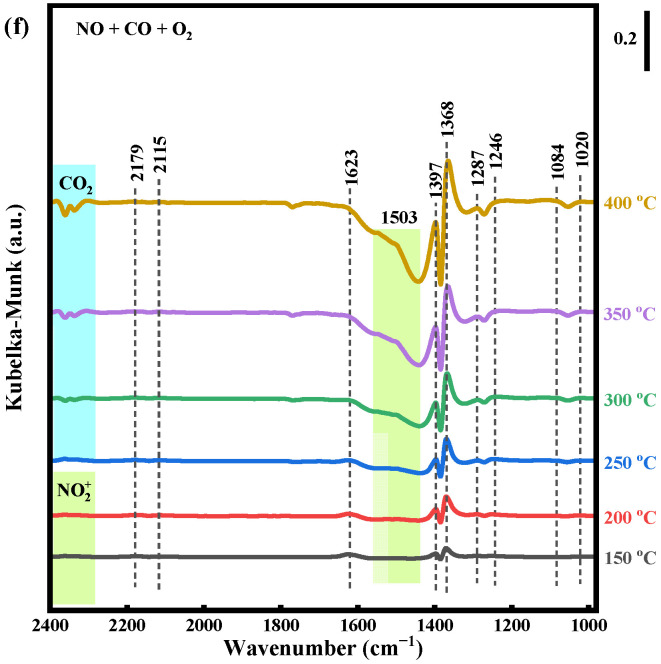
In situ DRIFTS spectra of CoCeOx-PVP catalyst from 150 to 400 °C. (**a**) NO adsorption, (**b**) NO + O_2_, (**c**) CO adsorption, (**d**) CO + O_2_, (**e**) NO + CO, (**f**) NO + CO + O_2_. (Reaction condition: [NO] = 500 ppm, [CO] = 3000 ppm, [O_2_] = 5 vol%, balanced with N_2_).

In order to investigate the interaction between the reactant gas and the CoCeOx catalyst, in situ DRIFTS under simulated reaction conditions is employed to analyze the species that are adsorbed on the surface, as shown in Figure 7.

#### 2.4.1. NO Adsorption and NO + O_2_ Reaction

In situ DRIFTS experiments of NO single adsorption are carried out at different temperatures (150–400 °C) to further investigate the interaction of NO with the CoCeOx-PVP catalyst, and the results are shown in Figure 7a, showing the in situ infrared diagram of single NO. The peaks at 1083 cm^−1^ and 1368 cm^−1^ are attributed to nitrite, which are generated by the strong interaction between the metal-active sites on the catalyst surface and the adsorbed NO species. The dense peak is observed at 1254 cm^−1^, which is attributed to the nitro (-${NO}_{2}$) bonding bands [39,54], indicating that surface ${NO}_{3}^{-}$ species will be converted to ${NO}_{2}$ species on the catalyst [62]. The absorption peak at 1026 cm^−1^ can be attributed to the infrared vibrations of the bridged bidentate nitrates [67]. The absorption peaks at 1287 and 1508 cm^−1^ could be attributed to the infrared vibrations of monodentate nitrates, and the peak at 1397 cm^−1^ belongs to the nitrate. The intensity of the peaks becomes more pronounced with increasing temperature. This indicated that more adsorbed NO molecules bind to cation sites on CoCeOx-PVP catalyst to form thermally stable nitrate species and do not readily decompose at high temperature [68]. The peaks at 1623 cm^−1^ belong to NO_2_ [29], the peak at 1742 cm^−1^ to N_2_O_4_, and the NO^+^ species appeared at 2380–2308 cm^−1^. It is worth mentioning that the oxidation of NO to NOx species (NO_2_, N_2_O_4_ and NO^+^ species) is relevant to surface adsorbed oxygen (O^−^ or $O_{2}^{2-}$, O^2−^). The more types of oxygen that are adsorbed on the surface, the more types of NOx species that are produced.

With the introduction of O_2_ (Figure 7b), the peak intensities of nitrate (located at 1397 cm^−1^) and nitrite (located at 1368 cm^−1^) significantly increase, with nitrite showing a particularly prominent enhancement, its intensity far exceeding that in the absence of O_2_. This phenomenon reveals that a large amount of NO adsorbed on the catalyst surface is directly oxidized by O_2_ into nitrate or nitrite. Additionally, the conversion of NO^+^ ions to ${NO}_{2}^{+}$ ions observed within the range of 2380–2308 cm^−1^ [69,70] may originate from the dissociation of NO_2_ or the oxidation of NO^+^ by O_2_ [71]. Notably, the peaks of monodentate nitrate (located at 1287 cm^−1^) and N_2_O_4_ (located at 1742 cm^−1^) disappear, while the peak of physically adsorbed NO_2_ is enhanced, confirming that NOx species (including N_2_O_4_ and ${NO}_{3}^{-}$) undergo dissociation in the presence of O_2_ to produce NO_2_. On the other hand, the peak intensity of nitro (located at 1254 cm^−1^) does not change with increasing temperature. Comparative in situ DRIFTS testing results of NO adsorption and NO + O_2_ indicate that, under oxygen conditions, NOx species on the surface of the CoCeOx-PVP catalyst primarily exist in the forms of nitrite and nitro.

Based on the above analysis, we have proposed the adsorption process of NO and the reaction mechanism of NO + O_2_ on the CoCeOx-PVP catalyst. Specifically, NO is first adsorbed onto the active sites of the CoCeOx-PVP catalyst (Equation (1)). Subsequently, these adsorbed NO molecules react with the abundant active oxygen species on the catalyst surface or the O_2_ in the surrounding atmosphere to form adsorbed NO_2_ (Equations (2) and (4)). Then, these adsorbed NO_2_ molecules undergo further dissociation, releasing gaseous NO_2_ (Equations (3) and (5)).

#### 2.4.2. CO Adsorption and CO + O_2_ Reaction

In situ DRIFTS spectra of CO single adsorption on the catalyst surface with increasing temperature from 150 to 400 °C are measured (Figure 7c). Two peaks at 2177 and 2112 cm^−1^ are observed, which are assigned to the positive branch (P) and refill branch (R) of gaseous CO [29]. The peaks observed at 2361–2338 cm^−1^ are the characteristic vibrations of physically adsorbed gaseous CO_2_, and the peak intensity becomes stronger with the increase in temperature, implying that the CO reacts with surface active oxygen species on the catalyst surface to generate more CO_2_ at a higher temperature. Moreover, as the reaction temperature increases, the intensity of CO is basically stable, suggesting that temperature had little effect on the adsorption capacity of gaseous CO, and the production of gaseous CO_2_ is generated by the transformation of other carbonate species [54]. The peak observed at 1747 cm^−1^ is attributed to carbonyl vibration. It may be that the unsaturated coordination interaction between CO and Co^3+^ ions leads to their reduction, while the subsequent CO adsorption in the reduction center produces Co^2+^-carbonyl species [29,72,73]. The peak at 1549 cm^−1^ is attributed to stretching vibrational modes of bidentate carbonates [39,65,74], and their intensities keep stable over 300 °C because of their good thermal stability in high-temperature conditions. The peaks at 1402 and 1246 cm^−1^ are attributed to monodentate carbonate, and the peak intensity at 1402 cm^−1^ increases with increasing temperature due to CO molecules and surface oxygen atoms being enhanced, and more monodentate carbonates are generated [54]. Two peaks observed at 1114 cm^−1^ and 1057 cm^−1^ correspond to the stretching vibration of the formed carbonates species. The infrared adsorption peaks at 1361 cm^−1^ could be attributed to symmetry vibrations of carboxyl (COO^−^), indicating that O^−^ or O^2−^ combined with CO molecules to produce the formate (-COOH) [64,75]. The peak intensity increases with temperature, suggesting that a large number of oxygen vacancies are formed on the surface of the CoCeOx-PVP catalyst. In addition, no absorption peak is detected at 150 °C, indicating that CO does not interact with the catalyst under 200 °C.

As shown in Figure 7d, compared to the case of CO adsorption, the in situ DRIFTS spectrum of CO + O_2_ exhibits a significant blue shift for all peak positions. This transformation may be attributed to the modification of surface oxygen species on the CoCeOx-PVP catalyst by O_2_. The altered active oxygen species interact with CO molecules, enhancing the stability of structures such as carbonates, carboxylates, and hydroxyls, which results in an increase in the wavenumber of the peaks. Furthermore, the intensity of all peaks, especially the bidentate carbonates at 1555 cm^−1^, the monodentate carbonates at 1250 cm^−1^, and the carbonyl peak at 1766 cm^−1^, is significantly higher than that during CO adsorption. This indicates that the presence of O_2_ significantly enhances CO oxidation. However, the physical adsorption peak of CO does not strengthen with increasing temperature, suggesting that most CO may directly participate in the reaction in a molecular state without forming CxOy species (such as carbonates, carbonyls) in O_2_-containing gas.

The adsorption process of CO and its reaction with O_2_ exhibit certain similarities to the adsorption of NO and its reaction with O_2_. Specifically, CO first adsorbs onto the active sites of the CoCeOx-PVP catalyst (Equation (6)). Subsequently, these adsorbed CO molecules are oxidized by the active oxygen on the catalyst surface or gaseous O_2_, converting them into CO_2_ (Equations (7) and (9)). Ultimately, the generated CO_2_ dissociates from the catalyst surface (Equations (8) and (10)).

#### 2.4.3. NO + CO and NO + CO + O_2_ Reaction

In situ DRIFTS spectra of NO + CO and NO + CO + O_2_ reactions on the CoCeOx-PVP catalyst are measured under simulated reaction conditions. For the NO + CO reaction, as illustrated in Figure 7e, the peak positions closely resembled those observed during the exclusive NO adsorption. Various NxOy species, including nitrates, nitrites, and nitrosyls, are observable, yet no vibrational peaks indicative of CxOy species, such as carbonates, carbonyls, and carboxylates, are detected. This finding suggests that, in the presence of both NO and CO, the catalyst surface exhibits a preferential adsorption for NO, thereby hindering the adsorption of CO, which is consistent with previous studies [29,39].

As the reaction temperature rises gradually, we observe CO_2_ adsorption peaks that are reminiscent of those seen during the CO adsorption process, but with notably greater peak heights and areas. This observation provides further confirmation that NO selective catalytic reduction by CO (CO-SCR) reaction indeed proceeds on the catalyst surface [76,77].

Upon introducing O_2_ into the NO + CO reaction system, in situ DRIFTS characteristics, as depicted in Figure 7f, largely mirrored those of the NO + CO reaction system. However, as the reaction temperature increases, the spectral characteristics display significant differences. Specifically, at temperatures below 250 °C, we observe peaks associated with ${NO}_{2}^{+}$, whereas at temperatures exceeding 250 °C, these peaks transition to CO_2_ peaks resulting from the CO + O_2_ reaction. This phenomenon underscores the simultaneous occurrence of NO oxidation, CO oxidation, and the CO-SCR reaction. At temperatures below 250 °C, the oxidation reactions of NO and CO predominate; however, at temperatures above 250 °C, the CO-SCR reaction emerges as the primary reaction, outpacing the oxidation reactions of NO and CO.

Co-OV-Ce + NO_(g)_ → Co-OV-Ce-NO_(ads)_ (1)

Co-OV-Ce-NO_(ads)_ + O^2−^/O^−^ → Co-OV-Ce-NO_2(ads)_ (2)

Co-OV-Ce-NO_2(ads)_ → Co-OV-Ce + NO_2(g)_ (3)

Co-OV-Ce-NO_(ads)_ + O_2_ → Co-O-Ce-NO_2(ads)_ (4)

Co-O-Ce-NO_2(ads)_ → Co-O-Ce + NO_2(g)_ (5)

Co-OV-Ce + CO_(g)_ → CO-OV-Ce-CO_(ads)_ (6)

Co-OV-Ce-CO + O^2−^/O^−^ → Co-OV-Ce-CO_2(ads)_ (7)

Co-OV-Ce-CO_2(ads)_ → Co-OV-Ce-CO + CO_2(g)_ (8)

Co-OV-Ce-CO_(ads)_ + O_2_ → Co-O-Ce-CO_2(ads)_ (9)

Co-O-Ce-CO_2(ads)_ → Co-O-Ce + CO_2(g)_ (10)

### 2.5. Proposed Reaction Mechanism

**Figure 8:**
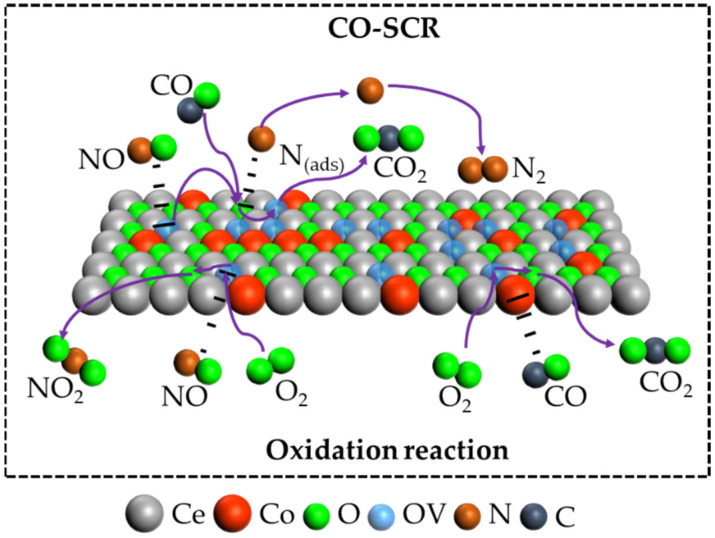
Schematic diagram of the CO-SCR reaction mechanism with CoCeOx-PVP catalyst in the presence of O_2_.

Based on the above analysis, the reaction pathway of the CoCeOx-PVP catalyst under O_2_-containing conditions is proposed as shown in Figure 8. In an environment where NO and CO coexist in the absence of O_2_, they engage in competitive adsorption on the catalyst surface. NO, with its unpaired electrons, has a higher affinity for occupying the oxygen vacancy interfaces of the catalyst (Equation (1)), thereby hindering the effective adsorption of CO. Subsequently, the N-O bond in NO undergoes dissociation, releasing O atoms that fill the oxygen vacancies and simultaneously forming adsorbed N atoms (Equation (11)). Throughout this process, CO directly participates in the reaction in its gaseous form, facilitating the regeneration of oxygen vacancies, accompanied by the release of N atoms and the formation of gaseous CO_2_ (Equation (12)). After that, on the surface of the CoCeOx-PVP catalyst, the second NO molecule reacts with the N atom, leading to the formation of N_2_O. Subsequently, this newly formed N_2_O undergoes dissociation at the oxygen vacancy sites of the catalyst, decomposing into N_2_ and O (Equation (13)) [29,39]. Finally, the second CO molecule reacts with O to generate the second CO_2_ molecule. This reaction mechanism aligns with the Eley–Rideal mechanism, where adsorbed NOx reacts with gaseous CO. This aligns with the earlier research conducted by our team’s Du Ying et al. on the NO-CO reaction catalyzed by the CuxOy-CeO_2_ multicomponent catalyst, which follows the Mars–van Krevelen (MvK) reaction mechanism. In this process, the formation of surface oxygen vacancies (SOVs) and surface synergistic oxygen vacancies (SSOVs) and the adsorption and dissociation of NO on oxygen vacancies played essential roles [78].

In the presence of O_2_ in the reactant gas, the oxidation reactions of NO and CO coexist with the CO-SCR reaction. Below 250 °C, the NO and CO adsorbed on the catalyst surface are more prone to oxidation by O_2_, converting them into NO_2_ and CO_2_, respectively (Equations (4), (5), (9) and (10)). This process consumes a significant number of oxygen vacancies, leading to a decrease in the activity of the CO-SCR reaction. However, as the temperature rises above 250 °C, the CO-SCR reaction becomes dominant and generates new oxygen vacancies, which not only enhances the adsorption of NO but also significantly boosts the activity of the CO-SCR reaction. It is important to note that, due to the much higher concentration of O_2_ compared to CO, as the reaction progresses, the oxygen vacancies will gradually be depleted, ultimately having an adverse impact on the progression of the CO-SCR reaction.

Co-OV-Ce-NO_(ads)_ → Co-O-Ce-N_(ads)_ (11)

Co-O-Ce-N_(ads)_ + CO → Co-OV-Ce + N + CO_2(g)_ (12)

N + NO_(ads)_ → N_2_O_(ads)_ → N_2(g)_ + O (13)

## 3. Materials and Methods

### 3.1. Materials

All the chemical reagents employed in this experiment were procured from reputable commercial suppliers and directly utilized without any additional purification processes. These include cobalt nitrate hexahydrate (Co(NO_3_)_2_·6H_2_O, analytical reagent grade, purity ≥ 99 wt%), cerium nitrate hexahydrate (Ce(NO_3_)_3_·6H_2_O, analytical reagent grade, purity ≥ 99 wt%), and polyvinyl pyrrolidone (PVP, K30, chemically pure reagent grade, purity ≥ 99 wt%) purchased from Aladdin Industrial Co., Ltd. (Shanghai, China). and sodium carbonate (Na_2_CO_3_, analytical reagent grade, purity ≥ 99.8 wt%) purchased from Sinopharm Chemical Reagent Co., Ltd. (Beijing, China). Throughout the experiments, deionized (DI) water was exclusively utilized.

### 3.2. Catalyst Preparation

**Scheme 1:**
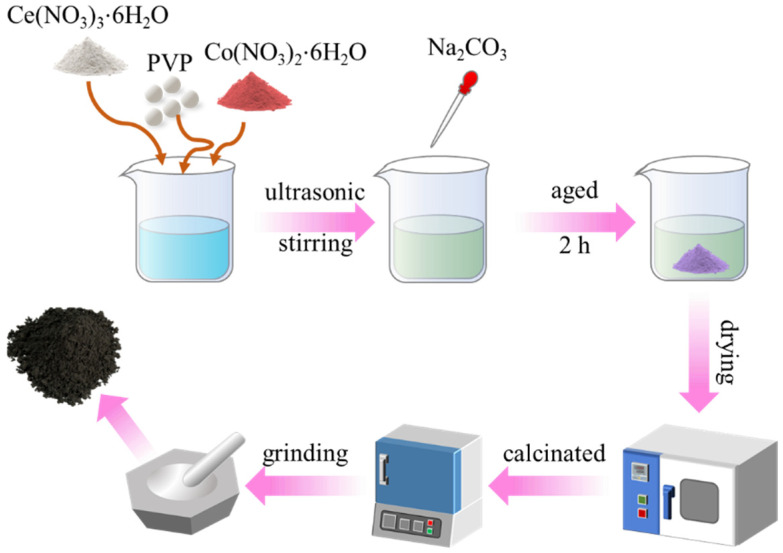
Schematic diagram of catalyst synthesis.

The synthesis procedure of the Co-Ce catalyst is illustrated in Scheme 1. Typically, the desired amounts of Co(NO_3_)_2_·6H_2_O and Ce(NO_3_)_3_·6H_2_O were individually dissolved in 100 mL of deionized (DI) water. Then, 10 mL of each nitrate solution was withdrawn, mixed with 10 g of PVP reagent, and the volume was adjusted to 150 mL with DI water. The resulting mixture underwent ultrasonic treatment for 30 min, followed by continuous stirring for 30 min. Subsequently, a 40 mL aliquot of Na_2_CO_3_ aqueous solution (1.25 M) was added dropwise while stirring continuously for 1 h. After allowing the mixture to age for 2 h, the formed carbonate precipitate was thoroughly washed three times with an excess of DI water and ethanol, followed by drying at 100 °C overnight. Finally, the dried solid was calcinated to 500 °C at a heating rate of 5 °C·min^−1^ in an air atmosphere and maintained at that temperature for 4 h to obtain the Co-Ce catalyst. The sample was pressed and ground to 40–60 mesh and ready for use.

### 3.3. Catalyst Characterization

The specific surface area was determined according to the Brunauer–Emmett–Teller (BET) method by nitrogen physisorption (ASAP 2020, Micromeritics, Norcross, GA, USA). The pore volume and pore size distribution were calculated by the Barett–Joyner–Halenda (BJH) method using the adsorption isotherm branch.

The microscopic structures of catalysts were characterized by field emission scanning electron microscopy (SEM) with a HITACHI SU8010 field emission scanning electron microscope.

The crystalline phases of the samples were measured by X-ray diffraction (XRD) using Co K*α* radiation (λ = 1.79026 Å), and the scanning step was 5°·min^−1^ from 10.0° to 90.0° at 40 kV and 40 mA (D8 Advance X, Bruker, Karlsruhe, Germany). The crystallite size of all samples was calculated using the Debye–Scherrer equation.

Raman spectra were acquired using a DXR2 (ThermoFisher Scientific, USA) instrument equipped with a 532 nm semiconductor laser.

The surface chemical compositions of the samples were determined by X-ray photoelectron spectroscopy (XPS) using monochrome Al K*α* X-ray radiation (*hv* = 1486.6 eV), with the carbon 1s peak (BE = 284.8 eV) used as the standard to calibrate all binding energies (Escalab 250Xi, ThermoFisher Scientific, Waltham, MA, USA).

The reducibility of the catalysts was investigated by hydrogen temperature-programmed reduction (H_2_-TPR) and experiments were carried out on an automated chemisorption analyzer (AutoChem II 2920, Micromeritics, GA, USA). Upon loading about 100 mg of one sample into a quartz U-tube, the sample was degassed at 200 °C for 1h and then naturally cooled to 50 °C under high-purity nitrogen (N_2_) gas flow (50 mL·min^−1^) and, subsequently, the flowing gas was changed to 10% H_2_/N. Finally, the sample was heated from 50 to 800 °C at 10 °C·min^−1^. The electrical signal was recorded by a thermal conductivity detector (TCD).

O_2_ temperature-programmed desorption (O_2_-TPD) experiments’ data were obtained by an automated chemisorption analyzer (AutoChem III, Micromeritics, GA, USA) with a thermal conductivity detector (TCD). The samples (50 mg) were pretreated at 400 °C for 60 min with 20% O_2_/N_2_ (30 mL∙min^−1^) and then exposed to 5% of O_2_/N_2_ for 60 min at 50 °C. Subsequently, the chamber was purged with N_2_ for 30 min. Finally, the temperature was increased to 800 °C under a N_2_ atmosphere at the rate of 10 °C∙min^−1^.

In situ DRIFTS experiments were performed on an FT-IR spectrometer (INVENIO-S, Bruker, Karlsruhe, Germany) equipped with a liquid nitrogen cooling detector (MCT) and a reaction cell (HVC-DRM-5, Harrick, Long Island, NY, USA) with ZnSe windows for the adsorption of NO and CO on catalysts. First, the catalyst sample and KBr were physically mixed in a weight ratio of 1:10 and pretreated in a flow of pure N_2_ (100 mL·min^−1^) at 400 °C for 1 h, then cooled down to the test temperature. The background spectra were collected during the cooling procedure in an atmosphere of pure N_2_ (100 mL·min^−1^) and subtracted from the corresponding spectra. During the DRIFTS tests, the adopted conditions were as follows: 500 ppm NO + 3000 ppm CO + 5 vol% O_2_, N_2_ as the balance gas, and a total flow rate of 100 mL·min^−1^. All these spectra were collected by accumulating 32 scans in the range of 400–4000 cm^−1^ at a resolution of 4 cm^−1^ as a function of time.

### 3.4. Catalytic Performance Evaluation

**Scheme 2:**
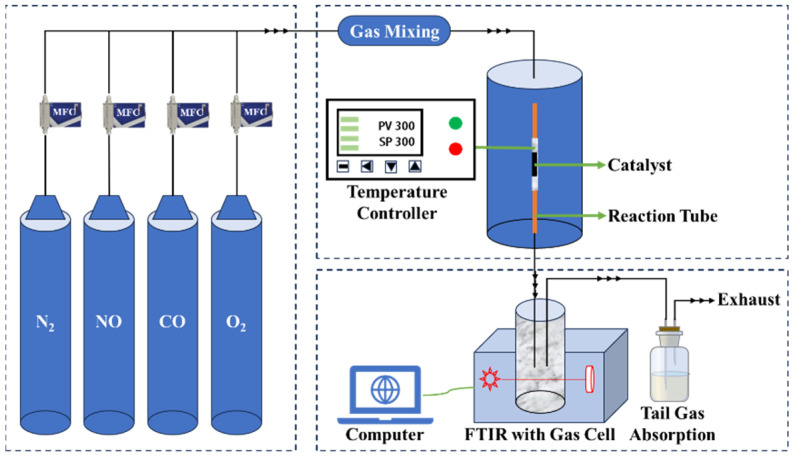
Schematic diagram of the experimental system.

The CO-SCR reaction was conducted in a fixed-bed quartz tubular reactor (inner diameter = 6 mm) with a thermocouple inserted into its center. The catalysts were treated in a CO/N_2_ (3000 ppm CO and N_2_ as balance gas) gas mixture at a flow of 500 mL·min^−1^ at 200 °C for 1 h before each test. The catalytic activity of each 20–40 mesh catalyst (about 400 mg, 0.3 mL) was examined, and experiments were performed at 150 to 400 °C. The inlet gas comprised NO (500 ppm), CO (3000 ppm), O_2_ (5 vol%), and N_2_ (the balance). The feed gas was introduced into the reactor at flow rate of 100 mL·min^−1^, corresponding to a gas hourly space velocity (GHSV) of 20,000 h^−1^. The gas concentration (NO, NO_2_, N_2_O, and CO) of each temperature point was analyzed in a stable state using an FT-IR spectrometer (INVENIO-S, Bruker, Germany) with a gas cell (2.4 m, PIKE, America) (Scheme 2). The NOx conversion (*ƞ_NOx_*), N_2_ selectivity (*S*_*N*2_), and CO conversion (*ƞ_CO_*) were calculated as the following equations:

(14) $$\eta_{NOx}=\frac{NOx_{in}-NOx_{out}}{NOx_{in}}\times 100\%$$

(15) $$S_{N2}(\%)=\left[1-\frac{2\times N_{2}O_{out}}{NOx_{in}-NOx_{out}}\right]\times 100\%$$

(16) $$\eta_{CO}=\frac{CO_{in}-CO_{out}}{CO_{in}}\times 100\%$$

where *NOx_in_* and *NOx_out_* are the volume fractions of NOx at the inlet and outlet, *CO_in_* and *CO_out_* are the volume fractions of CO at the inlet and outlet, and *N*_2_*O_out_* is the volume fraction at the outlet.

## 4. Conclusions

We have achieved successful synthesis of the CoCeOx-PVP catalyst, which effectively catalyzes the selective reduction of CO for NO in O_2_-containing environments through the incorporation of polyvinylpyrrolidone (PVP). The integration of PVP has refined the particle size and pore structure of the CoCeOx metal oxide, notably enlarging the pore diameters and consequently exposing a greater number of active sites, thereby augmenting the capacity to accommodate reactant molecules. These structural enhancements facilitate the creation of additional oxygen vacancies within the CoCeOx-PVP catalyst, enhancing its reducibility and significantly boosting its catalytic performance in the CO-SCR reaction.

Moreover, we conduct in situ DRIFTS experiments to delve into the reaction mechanism of CO catalyzing the reduction of NO over the CoCeOx-PVP catalyst in various atmospheres. The experimental findings reveal that the CO-SCR reaction adheres to the Eley–Rideal (E-R) mechanism, where gaseous CO catalyzes the reduction of adsorbed NO to produce N_2_, while simultaneously undergoing oxidation to CO_2_. In the presence of O_2_ in the reactant gas, the oxidation reactions of NO and CO coexist competitively with the CO-SCR reaction. It is only when the temperature exceeds 250 °C that the CO-SCR reaction becomes the predominant process. However, as the reaction progresses, the oxidation reactions regain prominence, impacting the catalyst’s activity.

Despite the fact that this research has not yet achieved sustained activity for CO-SCR under O_2_-containing conditions, we have made strides in delaying the deactivation rate of the catalyst. Our findings offer fresh perspectives and guidance for the advancement of non-noble metal catalysts in the selective catalytic reduction of NO by CO.

## Figures and Tables

**Table 1:** Pore structure properties of the catalysts.

Catalysts	BET Specific Surface Area ^a^ (m^2^/g)	Total Pore Volume ^b^ (cm^3^/g)	Average Pore Diameter ^b^ (nm)
CoCeOx-PVP	24.6	0.13	20.89
CoCeOx	54.3	0.20	14.37
CoOx	25.6	0.11	17.65
CeOx	135.2	0.09	2.56

^a^ Surface area determined by BET method. ^b^ Average pore diameter determined by BJH method.

**Table 2:** XRD results of the catalysts.

Catalysts	Crystallite Size ^a^ (nm)	Lattice Parameter ^a^ (Å)	Estimated Crystallite Size ^b^ (nm)	Strain ^c^ (%)
CoCeOx-PVP	9.4	5.4194	10.2	0.016
CoCeOx	5.2	5.4029	4.6	−0.367
CoOx	----	----	26.8	−0.022
CeOx	7	5.4141	9.8	0.506

^a^ Estimated from the XRD diffraction peak (2*θ* = 28.6°) for catalyst using the Debye–Scherrer equation. ^b^ Estimated crystallite size from peak broadening after fitting the profile. ^c^ Estimated strain from peak broadening after fitting the profile.

**Table 3:** Data Summary on XPS of the catalysts.

Catalyst	Co^2+^/Co (%)	Ce^3+^/Ce (%)	O_α_/O (%)	O_β_/O (%)
CoCeOx-PVP	49.58	43.37	44.57	53.14
CoCeOx	45.63	34.94	61.40	30.54
CoOx	36.44	----	43.13	44.61
CeOx	----	36.00	63.53	22.35

**Table 4:** The amounts of H_2_ consumption obtained by H_2_-TPR and O_2_ desorption by O_2_-TPD.

Catalysts	H_2_-TPR	O_2_-TPD
	Total H_2_ Consumption (mmol/g)	Total O_2_ Desorption (mmol/g)
CoCeOx-PVP	2.89	1.01
CoCeOx	3.10	0.66
CoOx	15.24	0.20
CeOx	0.47	0.93

**Table 5:** Comparison of catalytic performances between CoCeOx-PVP catalyst in this work and other transition non-precious metal catalysts.

Catalyst	Reaction Conditions				T_max_ (°C)	NO Conversion (%)	Ref.
	NO (ppm)	CO (ppm)	O_2_ (%)	GHSV (h^−1^)			
CoCeOx-PVP	1000	3000	5	20,000	250	100	This work
K/Cu/SmCe@TiO_2_	500	10,000	10	10,000	330	97	[56]
Cu-Ce-Fe-Co/TiO_2_	200	10,000	6	10,000	200	63	[57]
Co_SA_+CoOx_NC_/CZO	1000	2000	5	20,000	250	100	[58]
Co_3_O_4_-CeO_2_-IOV	1000	2000	5	20,000	250	100	[29]
Co_2.9_Cu_0.1_O_4_	50,000	50,000	2.5	8000	700	88	[59]
10% MnO_2_/TiO_2_	400	400	2	50,000	200	95	[60]
CuCoCe/2D-VMT	500	1000	1	102,000	300	100	[54]
Fe-Co/ASC	1000	5000	1	20,000	300	45	[61]
NiFe/CeO_2_	500	5000	0.5	22,800	225	100	[62]
Co_10_-CeO_2_	1000	15,000	0.1	30,000	350	93	[63]
H-CuCo-CeO_2_	500	1000	0	60,000	175	100	[39]
CuCe_0.2_Co_0.8_O_y_	1000	2000	0	50,000	175	100	[38]
Fe@CeO_2_-ZIF-8	500	1000	0	22,800	250	100	[64]
CoCeOx-OA	500	1000	2	80,000 mL/g∙h	400	50	[30]
Cu_0.15_Mn	50,000	100,000	0	24,000 mL/g∙h	275	100	[65]
Co-AR CeO_2_	50,000	50,000	0	60,000 mL/g∙h	300	100	[66]

## Data Availability

Data are available upon reasonable request.

## References

[1] Inomata Y., Kubota H., Hata S., Kiyonaga E., Morita K., Yoshida K., Sakaguchi N., Toyao T., Shimizu K.I., Ishikawa S. (2021). Bulk tungsten-substituted vanadium oxide for low-temperature NO_x_ removal in the presence of water. Nat. Commun..

[2] Andana T., Rappé K.G., Gao F., Szanyi J., Pereira-Hernandez X., Wang Y. (2021). Recent advances in hybrid metal oxide-zeolite catalysts for low-temperature selective catalytic reduction of NO_x_ by ammonia. Appl. Catal. B-Environ..

[3] Boyle E. (2017). Nitrogen pollution knows no bounds. Science.

[4] Anenberg S.C., Miller J., Injares R.M., Du L., Henze D.K., Lacey F., Malley C.S., Emberson L., Franco V., Klimont Z. (2017). Impacts and mitigation of excess diesel-related NO_x_ emissions in 11 major vehicle markets. Nature.

[5] Heo I., You Y.W., Lee J.H., Schmieg S.J., Yoon D., Kim C.H. (2020). Urealess NOx Reduction by Carbon Monoxide in Simulated Lean-Burn Exhausts. Environ. Sci. Technol..

[6] Leclercq B., Kluza J., Antherieu S., Sotty J., Alleman L.Y., Perdrix E., Loyens A., Coddeville P., Lo Guidice J.M., Marchetti P. (2018). Air pollution-derived PM2.5 impairs mitochondrial function in healthy and chronic obstructive pulmonary diseased human bronchial epithelial cells. Environ. Pollut..

[7] Lelieveld J., Evans J.S., Fnais M., Giannadaki D., Pozzer A. (2015). The contribution of outdoor air pollution sources to premature mortality on a global scale. Nature.

[8] Song J.H., Park D.C., You Y.-W., Kim Y.J., Kim S.M., Heo I., Kim D.H. (2020). Kinetic and DRIFTS studies of IrRu/Al2O3 catalysts for lean NO_x_ reduction by CO at low temperature. Catal. Sci. Technol..

[9] Gan L.N., Shi W.B., Li K.Z., Chen J.J., Peng Y., Li J.H. (2018). Synergistic Promotion Effect between NO_x_ and Chlorobenzene Removal on MnO_x_-CeO_2_ Catalyst. ACS Appl. Mater. Interfaces.

[10] Chen R.Y., Fang X.Y., Li J.H., Zhang Y., Liu Z.M. (2023). Mechanistic investigation of the enhanced SO_2_ resistance of Co-modified MnO_x_ catalyst for the selective catalytic reduction of NO_x_ by NH_3_. Chem. Eng. J..

[11] Imanaka N., Masui T. (2012). Advances in direct NO_x_ decomposition catalysts. Appl. Catal. A Gen..

[12] Boningari T., Pavani S.M., Ettireddy P.R., Chuang S.S.C., Smirniotis P.G. (2018). Mechanistic investigations on NO reduction with CO over Mn/TiO_2_ catalyst at low temperatures. Mol. Catal..

[13] Liu Z.Y., Zhou Y., Liu J., Chen J., Heck A.J.R., Wang F.J. (2019). Reductive methylation labeling, from quantitative to structural proteomics. TrAC-Trends Anal. Chem..

[14] Wang Y.H., Jiang Q.K., Xu L.L., Han Z.K., Guo S., Li G., Baiker A. (2021). Effect of the Configuration of Copper Oxide-Ceria Catalysts in NO Reduction with CO: Superior Performance of a Copper-Ceria Solid Solution. ACS Appl. Mater. Interfaces.

[15] Yao X.J., Yu Q., Ji Z.Y., Lv Y.Y., Cao Y., Tang C.J., Gao F., Dong L., Chen Y. (2013). A comparative study of different doped metal cations on the reduction, adsorption and activity of CuO/Ce_0.67_M_0.33_O_2_ (M = Zr^4+^, Sn^4+^, Ti^4+^) catalysts for NO plus CO reaction. Appl. Catal. B Environ..

[16] Carlotto S., Natile M.M., Glisenti A., Vittadini A. (2018). Catalytic Mechanisms of NO Reduction in a CO-NO Atmosphere at Co- and Cu-Doped SrTiO_3_(100) Surfaces. J. Phys. Chem. C.

[17] Xie X.W., Li Y., Liu Z.Q., Haruta M., Shen W.J. (2009). Low-temperature oxidation of CO catalysed by Co_3_O_4_ nanorods. Nature.

[18] Ma C.Y., Mu Z., Li J.J., Jin Y.G., Cheng J., Lu G.Q., Hao Z.P., Qiao S.Z. (2010). Mesoporous Co_3_O_4_ and Au/Co_3_O_4_ Catalysts for Low-Temperature Oxidation of Trace Ethylene. J. Am. Chem. Soc..

[19] Wang X.Y., Li X.Y., Mu J.C., Fan S.Y., Chen X., Wang L., Yin Z.F., Tadé M., Liu S.M. (2019). Oxygen Vacancy-rich Porous Co_3_O_4_ Nanosheets toward Boosted NO Reduction by CO and CO Oxidation: Insights into the Structure-Activity Relationship and Performance Enhancement Mechanism. ACS Appl. Mater. Interfaces.

[20] Wang L.Y., Cheng X.X., Wang Z.Q., Ma C.Y., Qin Y.K. (2017). Investigation on Fe-Co binary metal oxides supported on activated semi-coke for NO reduction by CO. Appl. Catal. B Environ..

[21] Zhang Z.L., Geng H.R., Zheng L.S., Du B. (2005). Characterization and catalytic activity for the NO decomposition and reduction by CO of nanosized Co_3_O_4_. J. Alloys Compd..

[22] Li H.F., Lu G.Z., Qiao D.S., Wang Y.Q., Guo Y., Guo Y.L. (2011). Catalytic Methane Combustion over Co_3_O_4_/CeO_2_ Composite Oxides Prepared by Modified Citrate Sol-Gel Method. Catal. Lett..

[23] Montini T., Melchionna M., Monai M., Fornasiero P. (2016). Fundamentals and Catalytic Applications of CeO_2_-Based Materials. Chem. Rev..

[24] Zhao B.M., Jian Y.F., Jiang Z.Y., Albilali R., He C. (2019). Revealing the unexpected promotion effect of EuO_x_ on Pt/CeO_2_ catalysts for catalytic combustion of toluene. Chin. J. Catal..

[25] Wang X., Zhao S., Zhang Y., Wang Z., Feng J., Song S., Zhang H. (2016). CeO_2_ nanowires self-inserted into porous Co_3_O_4_ frameworks as high-performance “noble metal free” hetero-catalysts. Chem. Sci..

[26] Deng C.S., Huang Q.Q., Zhu X.Y., Hu Q., Su W.L., Qian J.N., Dong L.H., Li B., Fan M.G., Liang C.Y. (2016). The influence of Mn-doped CeO_2_ on the activity of CuO/CeO_2_ in CO oxidation and NO plus CO model reaction. Appl. Surf. Sci..

[27] Deng Y.Q., Shi X.B., Wei L.Q., Liu H., Li J., Ou X.M., Dong L.H., Li B. (2021). Effect of intergrowth and coexistence CuO-CeO_2_ catalyst by grinding method application in the catalytic reduction of NO_x_ by CO. J. Alloys Compd..

[28] Savereide L., Nauert S.L., Roberts C.A., Notestein J.M. (2018). The effect of support morphology on CoO_x_/CeO_2_ catalysts for the reduction of NO by CO. J. Catal..

[29] Liu S.M., Xue W.J., Ji Y.J., Xu W.Q., Chen W.X., Jia L.H., Zhu T.Y., Zhong Z.Y., Xu G.W., Mei D.H. (2023). Interfacial oxygen vacancies at Co_3_O_4_-CeO_2_ heterointerfaces boost the catalytic reduction of NO by CO in the presence of O_2_. Appl. Catal. B Environ..

[30] Zeng J., Zhong X.M., Yu J., Zhang T., Wang Y.Z., Chang H.Z. (2020). Promotional Effect of Preparation Methods on Catalytic Reduction of NO by CO over CoCeO_x_ Catalysts. Ind. Eng. Chem. Res..

[31] Guo J., Zhang G.D., Tang Z.C., Zhang J.Y. (2020). Design of Prussian blue analogue-derived double-cone structure Ce-Fe catalysts and their enhanced performance for the selective catalytic reduction of NO_x_ with NH_3_. New J. Chem..

[32] Yan L.J., Liu Y.Y., Zha K.W., Li H.R., Shi L.Y., Zhang D.S. (2017). Scale Activity Relationship of MnO_x_-FeO_y_ Nanocage Catalysts Derived from Prussian Blue Analogues for Low-Temperature NO Reduction: Experimental and DFT Studies. ACS Appl. Mater. Interfaces.

[33] Passaro J., Imparato C., Parida D., Bifulco A., Branda F., Aronne A. (2022). Electrospinning of PVP-based ternary composites containing SiO_2_ nanoparticles and hybrid TiO_2_ microparticles with adsorbed superoxide radicals. Compos. Part. B Eng..

[34] Xu G.D., Song P., Xia L.X. (2024). Difunctional AuNPs@PVP with oxidase-like activity for SERRS detection of total antioxidant capacity. Talanta.

[35] Xue L., Zhang C.B., He H., Teraoka Y. (2007). Catalytic decomposition of N_2_O over CeO_2_ promoted CO_3_O_4_ spinel catalyst. Appl. Catal. B Environ..

[36] Yokota K., Fukui M., Tanaka T. (1997). Catalytic removal of nitric oxide with hydrogen and carbon monoxide in the presence of excess oxygen. Appl. Surf. Sci..

[37] Takahashi N., Shinjoh H., Iijima T., Suzuki T., Yamazaki K., Yokota K., Suzuki H., Miyoshi N., Matsumoto S., Tanizawa T. (1996). The new concept 3-way catalyst for automotive lean-burn engine: NO_x_ storage and reduction catalyst. Catal. Today.

[38] Wang X., Li X., Mu J., Fan S., Wang L., Gan G., Qin M., Li J., Li Z., Zhang D. (2019). Facile Design of Highly Effective CuCe_x_Co_1_–xO_y_ Catalysts with Diverse Surface/Interface Structures toward NO Reduction by CO at Low Temperatures. Ind. Eng. Chem. Res..

[39] He Y.J., Liu J., Zhang G.J., Wang Y., Zhao Y.Q., Li G.Q., Zhang Y.F., Lv D.K. (2023). Interfacial effects promote the catalytic performance of CuCoO_2_-CeO_2_ metal oxides for the selective reduction of NO by CO. Chem. Eng. J..

[40] Wang J., Xing Y., Jia H., Zhang W., Zhou H., Qian D., Su W. (2023). TiO_2_-coated CeCoOx-PVP catalysts derived from Prussian blue analogue for synergistic elimination of NOx and o-DCB: The coupling of redox and acidity. Chem. Eng. J..

[41] Lopes I., El Hassan N., Guerba H., Wallez G., Davidson A. (2006). Size-induced structural modifications affecting Co_3_O_4_ nanoparticles patterned in SBA-15 silicas. Chem. Mater..

[42] Huang J., Sheng H., Ross R.D., Han J., Wang X., Song B., Jin S. (2021). Modifying redox properties and local bonding of Co_3_O_4_ by CeO_2_ enhances oxygen evolution catalysis in acid. Nat. Commun..

[43] Kim J.H., Shin K., Kawashima K., Youn D.H., Lin J., Hong T.E., Liu Y., Wygant B.R., Wang J., Henkelman G. (2018). Enhanced Activity Promoted by CeO_x_ on a CoO_x_ Electrocatalyst for the Oxygen Evolution Reaction. ACS Catal..

[44] Qiu B.C., Wang C., Zhang N., Cai L.J., Xiong Y.J., Chai Y. (2019). CeO_2_-Induced Interfacial Co^2+^ Octahedral Sites and Oxygen Vacancies for Water Oxidation. ACS Catal..

[45] Ling T., Yan D.Y., Jiao Y., Wang H., Zheng Y., Zheng X.L., Mao J., Du X.W., Hu Z.P., Jaroniec M. (2016). Engineering surface atomic structure of single-crystal cobalt (II) oxide nanorods for superior electrocatalysis. Nat. Commun..

[46] Zhang X., Gao C., Wang Z., Wang X., Cheng J., Song X., Han X., Zhang N., Bao J., He G. (2022). Co_3_O_4_ with ordered pore structure derived from wood vessels for efficient Hg0 oxidation. Chin. J. Chem. Eng..

[47] Huang H., Dai Q.G., Wang X.Y. (2014). Morphology effect of Ru/CeO_2_ catalysts for the catalytic combustion of chlorobenzene. Appl. Catal. B Environ..

[48] Zhang Q.L., Liu X., Ning P., Song Z.X., Li H., Gu J.J. (2015). Enhanced performance in NO_x_ reduction by NH_3_ over a mesoporous Ce-Ti- MoOx catalyst stabilized by a carbon template. Catal. Sci. Technol..

[49] Bao T., Zhao Z.K., Dai Y.T., Lin X.L., Jin R.H., Wang G.R., Muhammad T. (2012). Supported Co_3_O_4_-CeO_2_ catalysts on modified activated carbon for CO preferential oxidation in H2-rich gases. Appl. Catal. B Environ..

[50] Wang J.Q., Shen M.Q., Wang J., Gao J.D., Ma J., Liu S.X. (2011). CeO_2_-CoO_x_ mixed oxides: Structural characteristics and dynamic storage/release capacity. Catal. Today.

[51] Jampaiah D., Venkataswamy P., Coyle V.E., Reddy B.M., Bhargava S.K. (2016). Low-temperature CO oxidation over manganese, cobalt, and nickel doped CeO_2_ nanorods. RSC Adv..

[52] Luo N., Gao F., Liu H., Xiong T., Wen J., Duan E., Wang C., Zhao S., Yi H., Tang X. (2024). Hierarchical structured Ti-doped CeO_2_ stabilized CoMn_2_O_4_ for enhancing the low-temperature NH3-SCR performance within highly H_2_O and SO_2_ resistance. Appl. Catal. B Environ..

[53] Liu S., Wu X., Tang J., Cui P., Jiang X., Chang C., Liu W., Gao Y., Li M., Weng D. (2017). An exploration of soot oxidation over CeO_2_-ZrO_2_ nanocubes: Do more surface oxygen vacancies benefit the reaction?. Catal. Today.

[54] Liu Z., Yu F., Pan K., Zhou X., Sun R., Tian J., Wan Y., Dan J., Dai B. (2021). Two-dimensional vermiculite carried CuCoCe catalysts for CO-SCR in the presence of O_2_ and H_2_O: Experimental and DFT calculation. Chem. Eng. J..

[55] Deng C.S., Li B., Dong L.H., Zhang F.Y., Fan M.G., Jin G.Z., Gao J.B., Gao L.W., Zhang F., Zhou X.P. (2015). NO reduction by CO over CuO supported on CeO_2_-doped TiO_2_: The effect of the amount of a few CeO_2_. Phys. Chem. Chem. Phys..

[56] Venegas F., López N., Sánchez-Calderón L., Aguila G., Araya P., Guo X., Zhu Y., Guerrero S. (2019). The transient reduction of NO with CO and naphthalene in the presence of oxygen using a core–shell SmCeO_2_@TiO_2_-supported copper catalyst. Catal. Sci. Technol..

[57] Pan K.L., Young C.W., Pan G.T., Chang M.B. (2020). Catalytic reduction of NO by CO with Cu-based and Mn-based catalysts. Catal. Today.

[58] Liu S.M., Ji Y.J., Liu B., Xu W.Q., Chen W.X., Yu J., Zhong Z.Y., Xu G.W., Zhu T.Y., Su F.B. (2023). Co Single Atoms and CoO_x_ Nanoclusters Anchored on Ce_0.75_Zr_0.25_O_2_ Synergistically Boosts the NO Reduction by CO. Adv. Funct. Mater..

[59] Li J., Luo G.H., Chu Y., Wei F. (2012). Experimental and modeling analysis of NO reduction by CO for a FCC regeneration process. Chem. Eng. J..

[60] Sreekanth P.M., Smirniotis P.G. (2008). Selective reduction of NO with CO over titania supported transition metal oxide catalysts. Catal. Lett..

[61] Cheng X., Wang L., Wang Z., Zhang M., Ma C. (2016). Catalytic Performance of NO Reduction by CO over Activated Semicoke Supported Fe/Co Catalysts. Ind. Eng. Chem. Res..

[62] Li S.H., Chen X.G., Wang F., Xie Z.L., Hao Z.R., Liu L.J., Shen B.X. (2022). Promotion effect of Ni doping on the oxygen resistance property of Fe/CeO catalyst for CO-SCR reaction: Activity test and mechanism investigation. J. Hazard. Mater..

[63] Zhang Z., Wang T., Pan X.H., Ma C.Y. (2020). Correlation of Physicochemical Characteristics of CoO_x_ Supported by a CeO_2_ Nanorod with NO Removal by CO. J. Environ. Eng..

[64] Li S.H., Wang F., Xie Z.L., Ng D., Shen B.X. (2023). A novel core-shell structured Fe@CeO_2_-ZIF-8 catalyst for the reduction of NO by CO. J. Catal..

[65] Liu T., Yao Y., Wei L., Shi Z., Han L., Yuan H., Li B., Dong L., Wang F., Sun C. (2017). Preparation and Evaluation of Copper Manganese Oxide as a High-Efficiency Catalyst for CO Oxidation and NO Reduction by CO. J. Phys. Chem. C.

[66] Zhang S., Lee J., Kim D.H., Kim T. (2020). NO reduction by CO over CoO_x_/CeO_2_ catalysts: Effect of support calcination temperature on activity. Mol. Catal..

[67] Wang J., You R., Qian K., Pan Y., Yang J., Huang W. (2021). Effect of the modification of alumina supports with chloride on the structure and catalytic performance of Ag/Al_2_O_3_ catalysts for the selective catalytic reduction of NO with propene and H_2_/propene. Chin. J. Catal..

[68] Liu T., Wei L., Yao Y., Dong L., Li B. (2021). La promoted CuO-MnO_x_ catalysts for optimizing SCR performance of NO with CO. Appl. Surf. Sci..

[69] Cheng X.X., Zhang X.Y., Su D.X., Wang Z.Q., Chang J.C., Ma C.Y. (2018). NO reduction by CO over copper catalyst supported on mixed CeO_2_ and Fe_2_O_3_: Catalyst design and activity test. Appl. Catal. B Environ..

[70] Hadjiivanov K.I. (2007). Identification of Neutral and Charged NxOy Surface Species of IR Spectroscopy. Catal. Rev..

[71] Liu J., Zang P.C., Liu X.Q., Mi J.X., Wang Y., Zhang G.J., Chen J.J., Zhang Y.F., Li J.H. (2022). A novel highly active catalyst form CuFeMg layered double oxides for the selective catalytic reduction of NO by CO. Fuel.

[72] Qi L., Yu Q., Dai Y., Tang C., Liu L., Zhang H., Gao F., Dong L., Chen Y. (2012). Influence of cerium precursors on the structure and reducibility of mesoporous CuO-CeO_2_ catalysts for CO oxidation. Appl. Catal. B Environ..

[73] Martínez-Arias A., Fernández-García M., Soria J., Conesa J.C. (1999). Spectroscopic Study of a Cu/CeO_2_ Catalyst Subjected to Redox Treatments in Carbon Monoxide and Oxygen. J. Catal..

[74] Dasireddy V., Likozar B. (2017). Selective catalytic reduction of NOx by CO over bimetallic transition metals supported by multi-walled carbon nanotubes (MWCNT). Chem. Eng. J..

[75] Zhao R.Q., Wei X.L., Chu B.X., Chen K.A., Qin Q.J., Liu H., Zhou Y.M., Li B., Dong L.H. (2022). Multi-phase coexisting metal oxide derived by MOFs for the CO-SCR reaction at low temperature and in situ DRIFTS study on reaction mechanism. Appl. Surf. Sci..

[76] Zhang Y., Zhao L., Kang M., Chen Z., Gao S., Hao H. (2021). Insights into high CO-SCR performance of CuCoAlO catalysts derived from LDH/MOFs composites and study of H_2_O/SO_2_ and alkali metal resistance. Chem. Eng. J..

[77] Savva P., Efstathiou A. (2008). The influence of reaction temperature on the chemical structure and surface concentration of active NOx in H_2_-SCR over Pt/MgOCeO_2_: SSITKA-DRIFTS and transient mass spectrometry studies. J. Catal..

[78] Du Y., Gao F., Tang X., Yi H., Zhou Y., Zhao S., Duan E., Wang J., Qi Z. (2023). Mechanistic insight into the enhanced NO reduction by CO over a pre-reduced Cu_x_O_y_-CeO_2_ multiphase catalyst. J. Environ. Chem. Eng..

